# Bioactive Phenolic Acid Derivatives and Undescribed Esculetin Glycosides from *Pseudopodospermum szowitzii* (DC.) Kuth.

**DOI:** 10.3390/molecules31091507

**Published:** 2026-05-01

**Authors:** Sezen Yılmaz Sarıaltın, Özlem Bahadır Acıkara, Büşra Yaylacı, Christian Zidorn

**Affiliations:** 1 Department of Pharmaceutical Toxicology, Faculty of Pharmacy, Ankara University, Tandogan, 06560 Ankara, Turkey; sezen.yilmaz@ankara.edu.tr; 2 Department of Pharmacognosy, Faculty of Pharmacy, Ankara University, Tandogan, 06560 Ankara, Turkey; byaylaci@ankara.edu.tr; 3 Abteilung Pharmazeutische Biologie, Pharmazeutisches Institut, Christian-Albrechts-Universität zu Kiel, Gutenbergstraße 76, 24118 Kiel, Germany; czidorn@pharmazie.uni-kiel.de; 4 Division of Pharmaceutical Biotechnology, Department of Pharmaceutical Biology and Biotechnology, Wroclaw Medical University, Borowska 211, 50-556 Wrocław, Poland

**Keywords:** antioxidant activity, Asteraceae, esculetin derivatives, *Pseudopodospermum szowitsii*, *Scorzonera mollis* subsp. *szowitsii*

## Abstract

*Pseudopodospermum szowitzii* (DC.) Kuth. a member of the Asteraceae family, grows naturally in the Irano-Turanian phytogeographical area, including Anatolia. In Anatolia, *P. szowitsii*, known as “goftigoda,” has edible young leaves and roots and is used in folk medicine for antidiabetic and analgesic properties. Nine compounds, including chlorogenic acid and derivatives from the ethyl acetate layer as well as 2,4,6-trimethoxyphenyl-1-*O*-*β*-glucopyranoside, 2,4,6-trimethoxyphenyl-1-*O*-*β*-apiofuranosyl-(1→6)-*β*-glucopyranoside, esculetin 6-*O*-*β*-glucopyranoside, and esculetin 6-*O*-*β*-apiofuranosyl-(1→6)-*β*-glucopyranoside from the water part of the methanolic extract, were isolated as known compounds. Notably, all esculetin derivatives have been isolated from the *Pseudopodospermum* for the first time, and among them, three compounds, esculetin 6-*O*-*β*-xylosyl-(1→6)-*β*-glucopyranoside, esculetin 6-*O*-*β*-glucopyranosyl-(1→3)-*β*-glucopyranoside, and esculetin 6-*O*-*β*-glucopyranosyl-(1→6)-*β*-glucopyranoside, were isolated as new esculetin heterosides that have not yet been isolated from any natural sources. The antioxidant activities of the total extract, phases, fractions, and compounds of *P. szowitsii* were also tested by evaluating their radical-scavenging capacities against DPPH and ABTS radicals. The ethyl acetate phase and the isolated compounds displayed significant antioxidant activity. The most active compound was caffeic acid, with IC_50_ values of 2.7 µg/mL and 3.4 µg/mL against DPPH and ABTS radicals, respectively, followed by dicaffeoylquinic acid derivatives and their methyl esters. On the other hand, none of the coumarin derivatives exhibited significant radical-scavenging activity.

## 1. Introduction

*Pseudopodospermum szowitzii* (DC.) Kuth., which is formerly recorded as *Scorzonera mollis* Bieb. subsp. *szowitsii* (DC) Chamberlain in the Flora of Turkey, is a perennial plant, naturally occurring in the Irano-Turanian phytogeographical area, including Armenia, the northwest part of Iran, Syria, and Türkiye, particularly in the central and eastern parts of Anatolia [1]. *P. szowitsii* is known as “goftigoda” and consumed as a vegetable, with its young leaves, roots, and aerial parts widely used in local cuisine [2,3]. *P. szowitsii* tubers are used to treat diabetes [4] and headaches [5] in Turkish folk medicine. Previous studies have revealed that *P. szowitsii* displayed wound healing and anti-inflammatory [6,7], antinociceptive [8], antihyperglycemic [9], antioxidant, cytotoxic, and enzyme-inhibitory activities [10].

Phytochemical investigations have revealed that phenolic compounds such as hyperoside, chlorogenic acid, rutin, luteolin-7-*O*-*β*-glucoside, gallic acid, protocatechuic acid, caffeic acid, *p*-coumaric acid, ferulic acid, quercetin, quercetin-3-*O*-*β*-xyloside, and chrysin were detected by HPLC and LC-MS/MS analysis [6,10]. Phenolic compounds are extensively studied bioactive molecules known for their significant health benefits, particularly their antioxidant properties. They act by donating hydrogen atoms or electrons to neutralize free radicals, and their activity largely depends on the number and position of hydroxyl groups in their structures [11]. Coumarin derivatives, including esculetin and its glucoside esculin, constitute a significant subclass of phenolic compounds, also recognized for their robust antioxidant potential. Extensive research has evidenced that these compounds can enhance the expression of key endogenous antioxidant enzymes, such as superoxide dismutase, glutathione peroxidase, and glutathione reductase [12].

Despite previous reports on the general phytochemical composition and biological activities of species within the Cichorieae tribe, the phytochemical profile of *Pseudopodospermum szowitsii* remains largely unexplored, particularly with respect to its polar constituents. To date, studies on this species have been limited, and there is no comprehensive investigation focusing on the isolation and structural characterization of secondary metabolites from its root extracts, especially from the aqueous fraction. In addition, although coumarin derivatives are known to be widely distributed within the Asteraceae family, information regarding esculetin glycosides in the genus *Pseudopodospermum* is extremely limited. Esculetin (6,7-dihydroxycoumarin) and its glycosides are of particular interest due to their reported antioxidant, anti-inflammatory, antidiabetic, and enzyme-inhibitory activities. However, the structural diversity, glycosylation patterns, and natural occurrence of esculetin derivatives in this genus have not yet been properly investigated.

Therefore, further phytochemical studies are required not only to reveal the chemical knowledge of *P. szowitsii* but also to identify novel coumarin glycosides with potential pharmacological relevance. In this context, the present study aims to investigate the secondary metabolites of *P. szowitsii*, with particular emphasis on the isolation and characterization of esculetin glycosides. Furthermore, to evaluate the potential antioxidant activities of these compounds using DPPH and ABTS radical-scavenging assays, and to identify previously undiscovered substances that possess significant antioxidant properties.

## 2. Results

### 2.1. Isolation and Structure Elucidation

**Figure 1:**
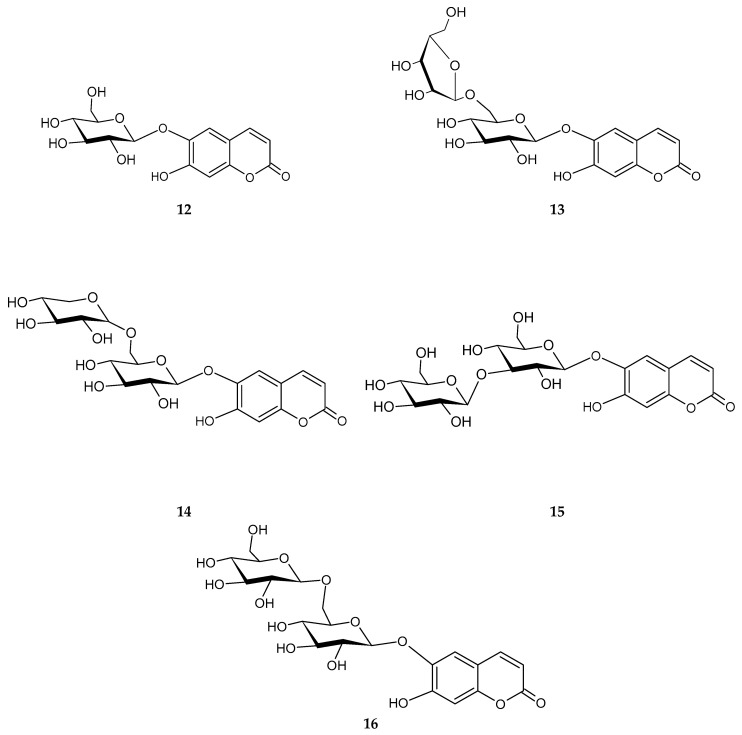
Chemical structures of the coumarin glycosides.

A methanolic root extract from *P. szowitsii* yielded nine and seven different compounds from the ethyl acetate and water layer, respectively. All isolated compounds from the ethyl acetate layer (SME), **1**–**9**, are known and have been previously isolated from other species. The structures of the compounds were established as protocatechuic acid (**1**) [13], caffeic acid (**2**), chlorogenic acid (**3**), chlorogenic acid methyl ester (**4**), 1,5-*O*-dicaffeoyl quinic acid (**5**), 3,5-*O*-dicaffeoyl quinic acid (**6**), 1,5-*O*-dicaffeoyl quinic acid methyl ester (**7**), 4,5-*O*-dicaffeoyl quinic acid methyl ester (**8**) [14,15,16], and scopolin (**9**) [17] based on their MS and NMR analysis results. The water layer of the methanolic extract gave seven different phenolic structures, which were identified as 2,4,6-trimethoxyphenyl-1-*O*-*β*-glucopyranoside (**10**), 2,4,6-trimethoxyphenyl-1-*O*-*β*-apiofuranosyl-(1→6)-*β*-glucopyranoside (**11**), esculetin 6-*O*-*β*-glucopyranoside (**12**), esculetin 6-*O*-*β*-apiofuranosyl-(1→6)-*β*-glucopyranoside (**13**), esculetin 6-*O*-*β*-xylosyl-(1→6)-*β*-glucopyranoside (**14**), esculetin 6-*O*-*β*-glucopyranosyl-(1→3)-*β*-glucopyranoside (**15**), and esculetin 6-*O*-*β*-glucopyranosyl-(1→6)-*β*-glucopyranoside (**16**), as shown in Figure 1. Compounds **10** and **11** were identified based on their MS and NMR values and comparison with the literature data as 2,4,6-trimethoxyphenyl-1-*O*-*β*-glucopyranoside and 2,4,6-trimethoxyphenyl-1-*O*-*β*-apiofuranosyl-(1→6)-*β*-glucopyranoside [18,19]. The structure of compound **12** was established as 6,7 dihydroxy coumarin-6-*O*-*β*- glucopyranoside, in other words, esculin [20,21], and **13** was identified as esculetin-6-*O*-*β*-D-apiofuranosyl-(1→6)-*O*-*β*-glucopyranoside [22].

**Figure 2:**
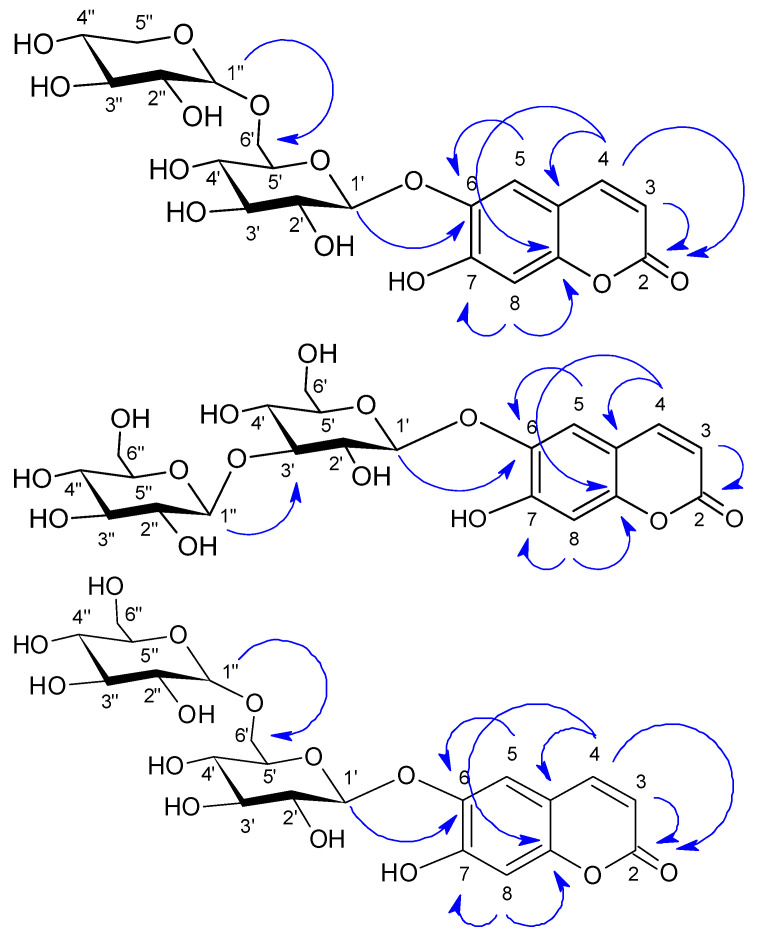
HMBC correlations of compounds **14**–**16**.

The compounds **14**–**16** were successfully isolated from the water layer of the plant and are especially remarkable, as they represent entirely novel structures that have not been previously reported from any natural source. All three compounds were observed on TLC under UV254 and UV366 light as light-blue and bluish-white fluorescent compounds, respectively. The compounds **14** and **15** were obtained as oily mixtures. In this mixture, compound **14** was the most abundant. The HPLC method was developed to separate the two coumarin derivatives; however, because they were minor components, only about 1 mg was obtained. The structures of compounds **14** and **15** were identified using NMR spectra, both in their separated and mixed forms (see Supplementary Materials). The molecular weight of compound **14** was calculated from HR-ESI-MS *m*/*z*: [M − H]^−^ for C_20_H_23_O_13_, 471.1144; found, 471.1172. All spectra of compound **14** were obtained, and a second compound **15** with a highly similar spectral profile was also detected. Despite their co-occurrence, differences in their relative abundance—compound **14** being the major constituent—allowed clear differentiation of all signals. Following separation, compound **14** was analyzed as a pure substance using NMR techniques, and comprehensive 1D and 2D data (HSQC and COSY) were successfully acquired. However, HMBC analysis of the pure compound did not provide sufficient information to fully establish the sugar linkage. Therefore, HMBC data obtained from the mixture were additionally used for structural elucidation (see Supplementary Material), and key correlations are presented in Figure 2.

The ^1^H-NMR spectrum of compound **14** exhibited two characteristic olefinic doublets at δ 6.1 (1H, d, *J* = 9.0 Hz, H-3) and 7.8 (1H, d, *J* = 9.6 Hz, H-4), consistent with an α-pyrone moiety, confirming a coumarin skeleton. In addition, two aromatic singlets at δ 6.7 (1H, s, H-8) and 7.3 (1H, s, H-5), supported by HSQC correlations with the carbons at δ 103.6 (C-8) and 114.7 (C-5), respectively, verified the existence of esculetin, substituted at C-6 and C-7 positions. Presence of glucose moiety was confirmed by anomeric proton δ 4.6 (d, *J* = 7.8 Hz, H-1′) and carbon signals δ 102.8 (C-1′) as well as δ 73.6 (C-2′), δ 76.1 (C-3′), δ 70.1 (C-4′), δ 76.1 (C-5′), δ 68.4 (C-6′) carbon and proton signals observed between δ 3.5–3.9 ppm. The C-6′ carbon signal of glucose, which was shifted to δ 68.4 ppm, suggested the linkage of xylose to glucose at C-6′. This connection was also confirmed from the HMBC spectra by observing the correlation between δ 3.9 (H-6′a), δ 3.5 (H-6′b) proton signals and δ 104.3 (C-1″) carbon signal as well as δ 4.1 (d, *J* = 9.0 Hz, H-1″) and δ 68.4 (C-6′). The existence of the xylose moiety was confirmed by a second anomeric proton signal placed at δ 4.1 (d, *J* = 9.0 Hz, H-1″) and anomeric carbon signal δ 104.3 (C-1″) together with 73.6 (C-2″), δ 76.8 (C-3″), δ 70.0 (C-4″), δ 66.1 (C-5″) carbon and proton signals observed between δ 2.9–4.1 ppm. All data obtained from COSY, HSQC, and HMBC spectra confirmed the presence of glucose and xylose. The glucose connection to the 6,7-dihydroxy coumarin cycle was determined by HMBC correlation between δ 142.7 (C-6) and δ 4.6 (d, *J* = 7.8 Hz, H-1′), indicating the C-6 position. Detailed analysis of the ^1^H and ^13^C NMR data of **14** using HSQC and HMBC spectra allowed complete structure assignment as esculetin 6-*O*-*β*-xylopyranosyl-(1→6)-*β*-glucopyranoside, which has not been reported from any natural source previously. On the other hand, compound **15** was also identified as an esculetin derivative by the presence of the two olefinic doublets at δ 5.8 (1H, d, *J* = 9.2 Hz, H-3) and 7.7 (1H, d, *J* = 9.1 Hz, H-4) together with two additional aromatic protons at δ 6.4 (1H, s, H-8) and 7.1 (1H, s, H-5) observed on the ^1^H-NMR spectrum. The ^13^C-NMR spectrum revealed that all carbon signals of compound **15** were placed very close to compound **14** carbon signals. All data are given in Table 1. However, it was clearly observed that the sugar moiety of compound **15** consists of two glucose molecules, and the linkage between them was quite different from that of compound **14**. Anomeric proton signals at δ 4.7 (d, *J* = 7.8 Hz, H-1′) and δ 4.6 (d, *J* = 8.4 Hz, H-1″), as well as anomeric carbon signals at δ 104.4 (C-1′) and δ 105.3 (C-1″), verified the presence of two glucose moieties. HMBC correlation between δ 148.9 (C-6) and δ 4.7 (d, *J* = 7.8 Hz, H-1′) as well as δ 86.9 ppm (C-3′) carbon signal and δ 4.6 (d, *J* = 8.4 Hz, H-1″) proton signal indicating glucose linkage to the aglycone part at C-6 position and second glucose moiety linkage at C-3′ position, respectively. As a result, compound **15** was structurally elucidated as esculetin 6-O-*β*-glucopyranosyl-(1→3)-*β*-glucopyranoside. Notably, this compound is reported here for the first time as a naturally occurring metabolite. Although it has previously been obtained as a semisynthetic derivative via transglycosylation of aesculin catalyzed by the *T. neapolitana* BglA (*β*-glucosidase) mutant [23]. This study decisively establishes the first isolation of the substance from a natural source, confirming its genuine natural origin.

All data are displayed in Table 1 and Table 2. The MS spectrum of the compound also confirmed the structure by the molecular weight, which was calculated and measured as HR-ESI-MS *m*/*z*: [M − H]^−^ calcd for C_21_H_25_O_14_, 501.1250; found 501.1321.

Compound **16**, also observed as blue fluorescent under UV_366_ light, indicated that the compound could also be a coumarin derivative similar to the previous compounds. From ^1^H NMR and ^13^C NMR spectra as shown in Table 1 and Table 2, all the data showed the presence of a 6,7-dihydroxy coumarin structure (two doublets observed at δ 7.8 ppm (*J* = 9.6 Hz, 1H) and δ 6.2 ppm (*J* = 9.6 Hz, 1H) and two singlets at δ 7.2 ppm and δ 6.8 ppm. Both ^1^H and ^13^C NMR data of this compound are similar to those of compound **14**; however, the sugar part has little difference. The mass spectrum showed the molecular weight of the compound, which was determined to be HR-ESI-MS *m*/*z*: [M + Na]^+^ calcd for C_21_H_26_O_14_Na, 525.1215; found, 525.1276. Two glucose moieties were confirmed by observing two anomeric proton signals at δ 4.4 (*J* = 7.6 Hz, 1H) ppm and δ 5.0 (*J* = 4.0 Hz, 1H) ppm as doublets from the ^1^H NMR spectrum and two anomeric carbon signals at δ 102.5 (C-1′) and δ 101.0 (C-1″) together with ten carbon signals placed between δ 75.3 and 60.6 ppm in the ^13^C NMR spectrum. Sugar connections to the esculetin and the other glucose were revealed by HMBC correlations between the δ 142.2 ppm (C-6) carbon signal and δ 4.4 (*J* = 7.6 Hz, 1H, H-1′) proton signal and between the δ 5.0 (*J* = 4.0 Hz, 1H, H-1″) proton signal and δ 68.1 ppm (C-6′) carbon signal (Figure 2). Based on the spectral data, compound **16** was identified as 6,7-dihydroxycoumarin-6-*O*-*β*-glucopyranosyl-(1→6)-*β*-glucopyranoside.

### 2.2. Antioxidant Activity

Scavenging activities on ABTS and DPPH free radicals of extracts, fractions, and compounds were examined to determine their antioxidant potential. Among *P. szowitsii* roots, methanolic extract and its fractions, which were obtained by liquid–liquid fractionation in different polarities, SME exhibited the highest DPPH free radical activity with IC_50_ values of 12.6 µg/mL, which was even more significant than the total methanolic extract of the roots. When compared for their inhibitory effects against ABTS free radicals, the chloroform fraction (SMC) showed the highest activity among the total methanol extract and its fractions, followed by the water phase of *P. szowitsii* (SMW) and SME fractions, respectively. Reference compounds generally showed higher ABTS and DPPH free radical-scavenging activities compared to the extracts in both assays (*p* < 0.05). However, the extracts with the highest activity, SME and SC, exhibited no significant difference from the reference compounds in each assay (*p* > 0.05). This indicates that the extracts exhibit high free radical-scavenging activity. Table 3 displays the results for the whole methanolic extract and its fractions.

To conduct further experiments, the SME phase was utilized, and all fractions obtained by column chromatography from the SME phase were tested for antioxidant properties. Among the SME fractions obtained from *P. szowitsii*, SME-2 was determined to be the most effective against ABTS free radicals with an IC_50_ value of 16.8 µg/mL, followed by SME-4 with 24.4 µg/mL. The order of scavenging activity of SME fractions is displayed in Table 4. SME-2 fraction also displayed the highest inhibitory activity against DPPH radicals with an IC_50_ value of 11.4 µg/mL. All the fractions of SME and their corresponding antioxidant activities are shown in Table 4.

The antioxidant activity of the SMW was investigated due to its unique phytochemical structure identified in previous studies. SMW demonstrated radical-scavenging activity against ABTS and DPPH radicals, with IC_50_ values of 37.7 µg/mL and 262.2 µg/mL, respectively. Among the fractions of SMW, SMW-D exhibited the strongest scavenging activity against ABTS free radicals, with an IC_50_ value of 64.5 µg/mL, followed by SMW-A, with an IC_50_ value of 123.5 µg/mL. SMW-A was more effective in suppressing oxidation induced by DPPH radicals, with an IC_50_ value of 86.9 µg/mL, as shown in Table 5.

Antioxidant activities of compounds isolated from SME and SMW are displayed in Table 6. The phenolic compounds that were subjected to testing displayed remarkable antioxidant activity against two radical species: ABTS and DPPH. The observed antioxidant effectiveness varied significantly based on the structural characteristics of the compounds. Among these, caffeic acid emerged as the most potent, demonstrating exceptional activity with IC_50_ values of 3.6 ± 0.3 μg/mL in the ABTS assay and 2.7 ± 0.2 μg/mL in the DPPH assay. Following closely were 1,5-*O*-dicaffeoyl quinic acid methyl ester, which showed antioxidant activities of 4.1 ± 0.0 μg/mL for ABTS and 2.3 ± 0.2 μg/mL for DPPH, along with 4,5-*O*-dicaffeoyl quinic acid methyl ester, which showed values of 5.0 ± 0.1 μg/mL and 2.8 ± 0.0 μg/mL, respectively. All of these compounds not only exhibited impressive potency but also surpassed the antioxidant capacities of the reference compounds used in the assays.

In the context of the ABTS assay, several compounds, including caffeic acid, chlorogenic acid, chlorogenic acid methyl ester, 1,5-*O*-dicaffeoyl quinic acid, 3,5-*O*-dicaffeoyl quinic acid, and their respective methyl esters, demonstrated activities comparable to that of Trolox, a well-known standard antioxidant compound. Interestingly, there were no statistically significant differences in the activities of these tested compounds compared with Trolox, as indicated by *p*-values greater than 0.05. Conversely, protocatechuic acid, scopolin, glycosides derived from esculetin, and trimethoxyphenyl glycosides displayed significantly lower antioxidant activity (*p* < 0.05), highlighting their lower effectiveness in scavenging radicals.

In the DPPH assay, the dicaffeoyl quinic acid derivatives, particularly the 3,5-*O*-dicaffeoyl quinic acid, exhibited strong radical-scavenging capabilities, with a measured activity of 3.4 ± 0.0 μg/mL. Notably, all major phenolic acids tested in this context showed significantly greater activity than BHT (butylated hydroxytoluene), a commonly used reference antioxidant, with *p*-values indicating a statistically significant difference (*p* < 0.05). Among the tested compounds, chlorogenic acid methyl ester showed comparatively weaker antioxidant activity, with IC_50_ values of 8.5 ± 0.0 μg/mL for ABTS and 8.4 ± 0.6 μg/mL for DPPH. Protocatechuic acid, meanwhile, consistently showed lower antioxidant capacity than the reference compounds in both assays, suggesting limitations in its efficacy.

## 3. Discussion

This study focused on *P. szowitsii* roots, consumed as a wild vegetable in Turkey. The antioxidant activity and phytochemical structures of the plant’s roots have been investigated. Notably, the ethyl acetate fraction obtained from the methanolic extract of the roots displayed the most significant capacity to neutralize. To identify the specific bioactive compounds responsible for this antioxidant activity, a detailed chromatographic analysis was performed on the ethyl acetate fraction. Each fraction obtained from this analysis was rigorously tested for antioxidant potential, enabling identification of the key active substances contributing to the extract’s overall efficacy. The isolated compounds were then evaluated for their ability to scavenge radicals, thus identifying the responsible compound(s). Nine known compounds were isolated; in both test models, caffeic acid had the highest activity. The second and third most active compounds were 1,5-*O*-dicaffeoyl quinic acid methyl ester and 4,5-*O*-dicaffeoyl quinic acid methyl ester, respectively.

Caffeic acid is one of the most extensively studied phenolic acids, commonly occurring in both medicinal plants and food sources, and is well recognized for its strong antioxidant properties. Experimental studies have demonstrated that caffeic acid exhibits concentration-dependent antioxidant activity across different assay systems. A significant reduction in DPPH radical levels was observed due to its scavenging capacity, reaching up to 93.9% inhibition at 20 μg/mL [24]. Similarly, its activity increased proportionally with concentration, from 5.7 ± 0.26% at 5 μg/mL to 28.5 ± 0.51% at 30 μg/mL, confirming a dose-dependent activity [25]. Based on linear regression analysis, the IC_50_ value for DPPH• scavenging was determined to be approximately 2.39 μg/mL, falling within the range of 0.5–5 μg/mL reported for effective antioxidants [26]. Caffeic acid clearly demonstrates a remarkable concentration-dependent capability to scavenge radicals, evidenced by an impressive IC_50_ value of 8.72 µg/mL in neutralizing DPPH free radicals [27]. This supports its role as an effective natural antioxidant, as seen in the current study with an IC_50_ of 2.7 ± 0.2 µg/mL. Potent antioxidant activity for ABTS•^+^ scavenging has also been observed by several studies. Caffeic acid has been reported to exhibit strong ABTS•^+^ radical-scavenging activity in a concentration-dependent manner. A significant reduction (*p* < 0.01) in ABTS•^+^ levels was observed within the 10–20 μg/mL range, confirming its effective scavenging capacity [24]. Similarly, its activity increased with concentration, rising from 9.5 ± 0.08% at 5 μg/mL to 32.1 ± 0.83% at 30 μg/mL [25]. Based on inhibition–concentration analysis, the IC_50_ value was determined as 1.96 μg/mL [26]. In addition, high scavenging activity (82.21 ± 0.86%) at low concentrations (IC_50_ 6.2 ± 1.2 µg/mL further supports its potent antioxidant potential and dose-dependent behaviour [27]. Various studies using different assay systems have consistently demonstrated the strong antioxidant activity of caffeic acid. Reported IC_50_ values include 0.37 μg/mL for SOD-like activity, 41.11 μg/mL for superoxide anion scavenging, 1.64 μg/mL for crocin bleaching, 18.17 μg/mL for hypochlorous acid scavenging, and 0.84–15.23 μg/mL for H_2_O_2_ scavenging. In addition, caffeic acid exhibited a high ferric-reducing capacity, with a FRAP value of 526.50 μM Fe(II)/μg at 50 μg/mL, further confirming its potent antioxidant potential [26,27]. In numerous instances, these values are comparable to, or even exceed, those of conventional antioxidants such as ascorbic acid and trolox. Additionally, caffeic acid offers further benefits, including greater chemical stability than ascorbic acid and its natural origin, which enhance its potential as a promising antioxidant agent. Its robust activity is primarily attributed to the presence of a catechol moiety, which facilitates efficient radical-scavenging and electron donation [26].

On the other hand, dicaffeoylquinic acid derivatives, as combined structures of quinic acid and chlorogenic acid, are widely found in plants. 1,5-*O*-dicaffeoyl quinic acid, as well as 3,5-*O*-dicaffeoyl quinic acid, were found to be in the roots of the *P. szowitsii* in relatively high amounts. 1,5-*O*-dicaffeoyl quinic acid exhibited significant antioxidant activity, with an IC_50_ value of 2.8 ± 0.1 µg/mL against the DPPH radical, higher than that of ascorbic acid (positive control) previously [28]. The antioxidant potential of 3,5-*O*-dicaffeoyl quinic acid was also determined using DPPH, ABTS radical scavenging, and FRAP assays. The compound showed appreciable DPPH (IC_50_ = 4.26 µg/mL) and ABTS (TEAC value = 0.9974) radical-scavenging activities, as well as FRAP activity (3.84 mmole of Trolox equivalent/g) [29]. According to the literature, dicaffeoylquinic acid derivatives (di-COQs) exhibit antioxidant activity through both direct (radical scavenging) and indirect (Fe^2+^-chelating) ways. The findings presented are further substantiated by structure–activity relationship studies, which indicate that the efficiency of antioxidants is markedly influenced by the presence, quantity, and positional arrangement of free phenolic hydroxyl groups on the aromatic ring. The obstruction or alteration of these hydroxyl groups significantly reduces antioxidant activity. Furthermore, the characteristics of the spacer connecting the carboxyl group to the aromatic ring are of considerable importance, with methylenic, ethylenic, and, particularly, unsaturated linkages enhancing antioxidant efficacy. These structural attributes facilitate improved electron delocalization and radical stabilization, thereby augmenting the overall antioxidant capacity of phenolic acids [28]. They showed dose-dependent scavenging of multiple radicals (DPPH•, ABTS•^+^, and PTIO•) via electron transfer (ET), hydrogen transfer (H^+^), and hydrogen atom transfer (HAT) pathways, with no evidence for a radical adduct formation (RAF) mechanism. Structure–activity analysis revealed that adjacent dicaffeoyl derivatives (e.g., 3,4- and 4,5-di-COQ) possess higher antioxidant and metal-chelating capacity due to the favourable spatial orientation of caffeoyl groups, whereas non-adjacent derivatives showed relatively lower redox activity [30]. Methyl esters of dicaffeoyl quinic acid exhibited significant antioxidant activity, which could be comparable to, but lower than, their non-methylated derivatives, such as the following. 3,5-*O*-dicaffeoylquinic acid displayed the highest antioxidant activity, followed by its methyl ester. In contrast, 3,5-*O*-dicaffeoylquinic acid demonstrated significantly stronger activity, with IC_50_ values of 3.97 ± 0.26 μg/mL and 3.74 ± 0.07 μg/mL, respectively, against ABTS and DPPH radicals, comparable to or better than the reference antioxidant trolox (5.42 ± 0.17 μg/mL). Similarly, 3,5-*O*-dicaffeoylquinic acid methyl ester showed potent antioxidant activity, particularly in ABTS radical-scavenging activity (IC_50_ = 3.09 ± 0.29 μg/mL), although its activity was reduced against the DPPH radicals (IC_50_ = 8.41 ± 0.52 μg/mL). In contrast, 4,5-*O*-dicaffeoylquinic acid exhibited moderate activity with IC_50_ values of 16.54 ± 0.55 μg/mL and 13.90 ± 1.14 μg/mL in the respective assays [15], which was not supported by the study of adjacent dicaffeoylquinic acid derivatives having higher antioxidant activity than the non-adjacent derivatives [30].

The water phase was analyzed comprehensively to identify noteworthy natural compounds. However, fractions obtained by various chromatographic techniques failed to demonstrate significant inhibitory activity against the DPPH and ABTS radicals. Additionally, a meticulous evaluation of the isolated compounds from the water phase revealed no notable antioxidant activity. Scopolin and esculetin 6-*O*-*β*-glucopyranoside, the only coumarin derivatives isolated, exhibited notably weak antioxidant activity in both assays. Scopolin showed moderate activity with an IC_50_ value of 36.1 ± 5.4 μg/mL, whereas esculetin 6-*O*-*β*-glucopyranoside displayed considerably lower activity (IC_50_ = 67.6 ± 4.36 μg/mL) in the ABTS radical-scavenging assay. In the DPPH assay, neither compound reached 50% inhibition at the tested concentrations (IC_50_ > 100 μg/mL), indicating very limited radical-scavenging capacity. These results are supported by the literature, which reports that esculetin 6-*O*-*β*-glucopyranoside has been widely shown to exhibit strong antioxidant activity through both direct free radical-scavenging and modulation of cellular defence systems. It effectively scavenges various radicals, including O_2_•^−^, NO•, and DPPH, with reported IC_50_ values of 69.27 μg/mL, 8.56 μg/mL, and 0.141 μM, respectively. Mechanistically, esculetin 6-*O*-*β*-glucopyranoside activates the Nrf2 signalling pathway by disrupting the Keap1–Nrf2 interaction, as demonstrated by several studies. This disruption leads to upregulation of endogenous antioxidant enzymes, including superoxide dismutase (SOD), catalase (CAT), glutathione peroxidase (GPx), and glutathione reductase (GR). Both in vitro and in vivo studies have demonstrated that esculetin 6-*O*-*β*-glucopyranoside effectively reduces reactive oxygen species (ROS) accumulation and protects against oxidative damage across various experimental models [12]. Esculetin (6,7-dihydroxycoumarin) as the aglycone of esculin generally exhibits stronger antioxidant activity than scopoletin (6-methoxy-7-hydroxycoumarin), mainly due to the presence of an ortho-dihydroxyl (catechol) moiety, which enhances electron donation, radical stabilization, and metal chelation [31,32], and esculin as the sugar moiety masks the phenolic hydroxyl groups, reducing their ability to donate hydrogen atoms or electrons [33,34]. In contrast, methoxylation of scopoletin reduces the number of free hydroxyl groups, thereby diminishing its radical-scavenging capacity. Glycosylation further decreases the antioxidant activity of coumarin derivatives, which possess substantially lower antioxidant activity than more active phenolic constituents, likely due to the masking of free hydroxyl groups by sugar moieties [12,31,32].

Analysis of the isolated compounds revealed that the phenolic compounds exhibited significantly greater radical-scavenging capacity, indicating more potent antioxidant activity than the coumarins. The fundamental difference in the antioxidant activities of phenolics and coumarins stems from their basic skeletal structures. Phenolic acids, which form the core structure of phenolics, are compounds with a carboxylic acid structure derived from benzoic or cinnamic acid skeletons, and their structures consist of a phenolic ring with a carboxylic acid group [35]. Coumarins are oxygen-containing, organic heterocyclic compounds that possess a benzo-α-pyrone skeleton formed by the fusion of benzene and pyrone rings [36]. The structural flexibility of phenolic acids facilitates their extensive integration across various industrial applications. Due to their high flexibility, stability, and antioxidant capacity, these compounds are incorporated into formulations designed to enhance flexibility and durability by forming hydrogen bonds with biopolymer chains such as zein or cellulose [37,38]. Due to the coumarin scaffold being an aromatic bicyclic structure that acts as a cyclic lactone, containing one ring oxygen and one external oxygen atom, the structure is more rigid and less flexible than that of phenolic acids [39]. The antioxidant activity of plant-derived compounds is strongly influenced by their structural features, particularly the number and position of hydroxyl groups. An increase in hydroxyl group density enhances the ability of these compounds to donate hydrogen atoms or electrons, thereby improving their capacity to neutralize free radicals. Polyhydroxylated compounds generally exhibit higher antioxidant activity than monohydroxylated analogues, as the presence of multiple hydroxyl groups facilitates greater radical stabilization through resonance. In addition, conjugated double-bond systems further support electron delocalization after hydrogen donation, increasing overall antioxidant efficiency. Functional groups such as phenolic hydroxyls and, to a lesser extent, methoxy groups contribute to this activity, although free hydroxyl groups remain the most critical determinants.

In this context, phenolic acids such as caffeic acid and its derivatives typically display stronger antioxidant activity than coumarin-based compounds, largely due to their free catechol structure and conjugated side chains, which enable efficient radical-scavenging and electron delocalization [26,40]. Furthermore, dicaffeoylquinic acid derivatives exhibit enhanced activity compared to mono-caffeoyl analogues, reflecting the additive effect of multiple hydroxylated aromatic moieties [41]. Accordingly, antioxidant potency generally follows the trend: polyhydroxylated phenolic acids ≥ aglycone coumarins (particularly esculetin) > methoxylated coumarins (e.g., scopoletin) > glycosylated derivatives, emphasizing the importance of free hydroxyl groups, conjugation, and the absence of glycosylation in maximizing antioxidant activity.

From a chemical perspective, some of the isolated compounds from the water phase were interesting because they had not been previously isolated from any natural sources. In the current study, five esculetin glycosides were isolated. According to the literature, compounds **12** and **13** were isolated for the first time from *Pseudopodospermum szowitzii*, while compounds **14**–**16** have not been previously isolated from any natural sources containing esculetin as the aglycone, together with unidentified sugar moieties. Esculetin, 6,7-dihydroxycoumarin, is widely distributed in nature, including *Aesculus hippocastanum* L. as well as *Fraxinus rhynchophylla* Hance, which are the best-known sources [42]. In addition, from the Asteraceae family, *Taraxacum formosanum* Kitam. [43], *Taraxacum mongolicum* [44], *Aster yomena* [45], *Arnoseris minima* [46], *Solidago caucasica* [47], *Achillea millefolium* [48], *Artemisia capillaris* var. *acaulis* Pamp. [42], and *Cichorium* sp. [49] was reported as a natural source of esculetin and its glycosides.

Although simple coumarin derivatives such as umbelliferone, esculetin, and scopoletin are commonly found in all angiosperms, particular families including Apiaceae, Asteraceae, Fabaceae, Moraceae, Oleaceae, Rutaceae, and Thymelaeaceae are known as their most important sources [50,51]. Esculetin is a widely found coumarin derivative in members of the Cichorieae, such as *Cichorium*, *Pilosella*, and *Taraxacum*. The genus *Scorzonera* is also a member of the Cichorieae tribe of the Asteraceae family [45]. *Scorzonera undulata* ssp. *deliciosa*, *S. cana* var. *jacquiniana* (*Podospermum canum*) contains the 7-*O*-glycoside derivative of esculetin (cichoriin) [52]. Scopoletin and scopolin, glycosidic forms of esculetin methoxy derivative, have been isolated from certain *Scorzonera* species such as *S. divaricata* Turcz. [53,54], *S. pseudivaricata* [53], and *S. alexandrina* [55].

## 4. Conclusions

This study provides a comprehensive phytochemical and biological evaluation of the roots of *Pseudopodospermum szowitsii*, emphasizing significant antioxidant activity and notable structural diversity among compounds. The ethyl acetate fraction was the most bioactive, showing strong radical-scavenging activity, mainly linked to phenolic acids, especially caffeic acid and various dicaffeoylquinic acid derivatives, which, in some cases, surpassed the effectiveness of standard antioxidants.

In total, sixteen compounds were isolated and characterized structurally, including a notable series of coumarin glycosides. Among these, three new esculetin derivatives were identified for the first time in nature: esculetin 6-*O*-*β*-xylosyl-(1→6)-*β*-glucopyranoside, esculetin 6-*O*-*β*-glucopyranosyl-(1→3)-*β*-glucopyranoside, and esculetin 6-*O*-*β*-glucopyranosyl-(1→6)-*β*-glucopyranoside. The presence of multiple esculetin glycosides in this species highlights its unique chemotaxonomic significance within the tribe Cichorieae.

While the present study provides valuable insights into the phytochemical profile and antioxidant potential of *P. szowitsii*, certain limitations should be noted. The antioxidant activity was primarily evaluated using in vitro assays (ABTS and DPPH), which, although widely accepted, may not fully represent biological performance under physiological conditions. In addition, in vivo validation was not within the scope of the current study, and the biological evaluation of the isolated compounds, particularly the newly identified esculetin glycosides, was primarily limited to antioxidant assays. Nevertheless, these aspects do not diminish the significance of the structural findings and the observed bioactivity trends, but rather highlight areas for further investigation.

Future research should prioritize in vivo studies to validate the biological significance of the extracts and their active constituents. The structural diversity and novelty of the isolated coumarin glycosides, particularly the newly identified esculetin derivatives, position these compounds as promising candidates for further biological screening, including investigations into their anti-inflammatory and antidiabetic properties. Moreover, mechanistic studies are essential to elucidate their molecular modes of action. It is also important to focus on the bioavailability, metabolism, and pharmacokinetic behaviour of glycosylated compounds, as glycosylation is likely to play a pivotal role in modulating their biological efficacy. Ultimately, these research directions will be crucial for translating the current findings into potential therapeutic applications.

In conclusion, *P. szowitsii* has been identified as a noteworthy natural source of bioactive compounds, distinguished by its strong antioxidant properties and the presence of structurally unique secondary metabolites. The findings of this study not only advance the chemical characterization of this species but also establish a basis for future pharmacological and mechanistic investigations concerning the newly identified esculetin glycosides.

## 5. Materials and Methods

### 5.1. Plant Material

**Figure 3:**
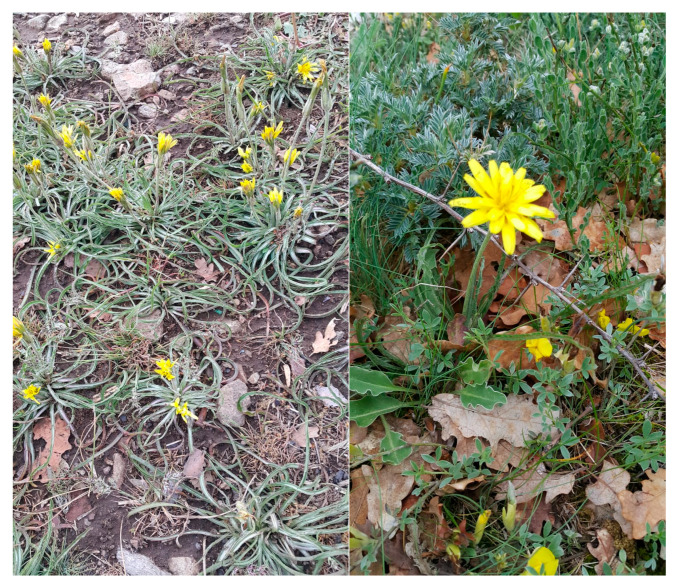
*Pseudopodospermum szowitsii* (DC.) Kuth. (Photo by Ö.Bahadır-Acıkara).

*Pseudopodospermum szowitzii* (DC.) Kuth. (Syn. *S. mollis* Bieb. ssp. *szowitzii* (DC.) Chamb.) (the species was formerly also known as *Scorzonera mollis* subsp. *szowitsii* and is recorded in the Flora of Turkey) was collected at N 40°26′49.1″ and E 32°37′6.3″ Kızılcahamam–Ankara, Türkiye (A4), in May 2020. The plant (Figure 3) was identified by Professor Hayri Duman from Gazi University, Faculty of Science, Department of Biology. The voucher specimen was deposited in Ankara University Faculty of Pharmacy Herbarium (AEF 30730).

### 5.2. Extraction and Isolation

An extract was prepared from dried and pulverized roots of *P. szowitsii* (400 g) by maceration at room temperature for 24 h, followed by one-hour extraction in an ultrasonıc bath (Bandelin Electronic DT 510 H (Germany-Berlin), 35 kHz, 160 W, at room temperature) using methanol five times. The methanolic extract was filtered and evaporated under vacuum to dryness. Liquid–liquid extraction was applied to obtain fractions in different polarities. For this purpose, the crude methanolic extract (66.8 g) was suspended in water (500 mL) and successively extracted with n-hexane, chloroform, and ethyl acetate in equal volumes (×4 times), yielding four layers of different polarity, along with the remaining water fraction of the methanolic extract. Ethyl acetate and water phases were subjected to further studies of antioxidant activity. Initially, the separation of the ethyl acetate phase (SME) (4.68 g) was performed using a Flash Chromatography system (Büchi, Switzerland-Flawil) on a reversed-phase (LiChroprep 25–40 µm C-18, Darmstadt-Germany) column (36 mm × 920 mm) with a methanol:water gradient from 10% to 100% at 20 mL/min, yielding 86 fractions of 50 mL each. All fractions were analyzed to reveal their phytochemical profiles by TLC on silica gel 60 precoated plates (Merck 5554, Darmstadt-Germany) using EtOAc:MeOH:water (100:13.5:10). Fractions were combined according to their phytochemical profiles into 12 main fractions, numbered SME-1 to SME-12. According to the antioxidant activity results, SME-2 was subjected to further purification and isolation procedures to obtain pure active compounds. Sephadex LH-20 (25–100 μm, Sigma-Aldrich, Steinheim, Germany) in a column (35 mm × 600 mm) was used to separate the SME-2 fraction, which allowed 49 subfractions. Compounds **1**–**3**, **5** and **6** were purified by preparative TLC on reverse-phase TLC plates (Merck 5559, Darmstadt-Germany) using MeOH:Water (1:1) as the solvent system. Compounds **4**, **7**–**9** were also obtained using the aforementioned method with MeOH:Water (8:2) as the solvent system. All structures were established based on their ^13^C and ^1^H NMR values and MS analyses.

**Figure 4:**
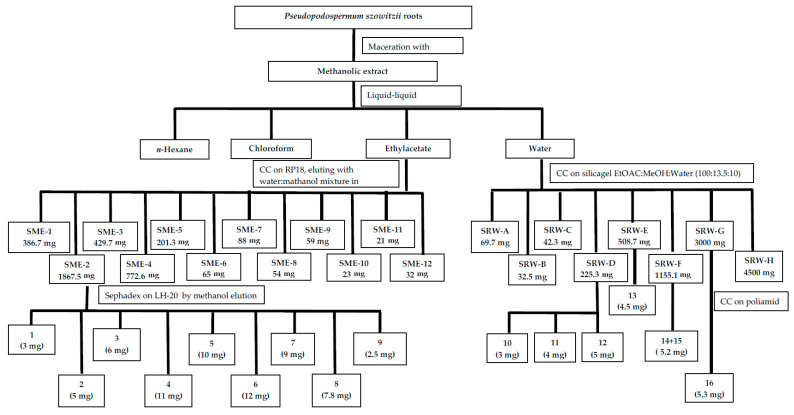
Isolation of the compounds from *P. szowitsii* root.

Preliminary TLC analysis of the water phase (SMW) from *P. szowitsii* root extract indicated that the water phase contains unusual chemical structures. In particular, several compounds, which were visible under UV254 and UV366 light on TLC as light-blue and bluish-white fluorescing spots, respectively, were tentatively identified as coumarin derivatives that could be of interest, given their limited distribution in the Asteraceae family. The SMW (17.8 g) phase was separated on silica gel by column chromatography using EtOAc:MeOH:water (100:13.5:10) as the solvent system, which was determined experimentally to be the most suitable. Eight main fractions named Fr WA-WH were obtained. All fractions were investigated for their phytochemical profiles by TLC on silica gel F254 (Merck, 1. 05554 20 × 20, Darmstadt-Germany) precoated plates using two different solvent systems made up EtOAc:MeOH:water (100:13.5:10) and CH_2_Cl_2_:MeOH:Water (40:12.5:4), then the plates were examined under UV light, and Vanillin-H_2_SO_4_ reagent was used for detection. SMW fractions were found to contain several interesting structures mentioned above, as well as a high amount of sugar derivatives. According to the antioxidant activity results, neither the SMW nor the fractions obtained from this phase displayed notable activity compared to references against both ABTS and DPPH radicals. However, the strongest inhibition of the ABTS radical was observed upon treatment with SMW-D. To isolate compounds **10**–**12**, SMW-D (225 mg) was loaded onto a Sephadex LH-20 column and eluted with methanol. Compounds **10** and **11** were not observed under UV light, and vanillin-H_2_SO_4_ reagent was used for detection. Further purification of these compounds from Sephadex fractions was performed by preparative TLC using EtOAc:MeOH:water (100:13.5:10) as a solvent system. Compound **12** was obtained from the 22–27 Sephadex fractions of SMW-D by semi-prep HPLC on an ACE 5 C18 HPLC column (Scotland, Aberdeen) using a mobile phase consisting of ACN and water mixture in a gradient system. Compound **13** and a mixture of **14** and **15** were also obtained from SMW-E (508.70 mg) and SMW-F (1151.50 mg), respectively, using separation on Sephadex LH-20 column chromatography, which was followed by semi-preparative HPLC on an ACE-RP-C18 (250 mm × 4.6 µm, 5 µ) column and elution of an ACN:water mixture in decreasing polarity as follows: 0th min ACN 5% and water 95%, changed to the ACN 15% and water 85% in 30th min at 3 mL/min flow rate, 210 nm. Compound **13** was isolated to sufficient purity for structure elucidation, whereas it was unable to separate compounds **14** and **15**. Compounds **14** and **15** gave two peaks very close to each other on HPLC, whereas no distinct spots were observed on TLC. On the other hand, NMR spectra successfully identified the structures of both compounds in a mixture. Compound **16** was isolated from SMW-G (3780 mg) by chromatography on a polyamide column eluted with water containing methanol in increasing amounts. Compound **16** was successfully isolated from 13 to 15 fractions on the polyamide column (filled with polyamide 60, 20 mm × 200 mm) by eluting with only water (100%). All procedures used to obtain compounds from *P. szowitsii* methanolic extract were summarized in Figure 4 for both water and ethyl acetate phases.

### 5.3. Structure Elucidation

1D (^1^H NMR, ^13^C NMR) and 2D (HSQC, HMBC, COSY) NMR spectra were recorded on a Bruker Avance III 400 NMR spectrometer operating at 400 MHz for the proton channel and 100 MHz for the ^13^C channel (Bruker BioSpin GmbH, Rheinstetten, Germany) in CD_3_OD, DMSO-*d*_6_, and D_2_O. Reference values were 2.50 (^1^H) and 40.12 ppm (^13^C) for dimethyl sulfoxide, 3.31 (^1^H) and 49.15 ppm (^13^C) for MeOH, and 4.75 (^1^H) for water, respectively. HR-MS analysis was carried out using Bruker Daltonics: maXis II ETD nLC/LC-QTOF-Mass Spectrometer (Germany, Bremen).

### 5.4. In Vitro Antioxidant Activity

#### 5.4.1. DPPH Free Radical-Scavenging Activity

Plant extracts and compounds were tested for their ability to remove DPPH free radicals by measuring their decolorization potential [56]. Firstly, a 100 μM solution of DPPH was prepared. Samples at varying concentrations were mixed with the 100 μM DPPH solution to a final volume ratio of 1:10 (*v*/*v*). Final concentrations were 10, 25, 50, 100, 250, and 500 µg/mL for the extracts and 1, 2.5, 5, 10, 25, 50, and 100 µg/mL for the compounds. The mixture was incubated at room temperature in the dark for 30 min. Then, the absorbance was measured at 517 nm using the SpectraMax^®^ 190 Microplate Reader (Germany-Ortenberg) at room temperature. This measurement was used to calculate the scavenging activity of the mixture, which is a measure of its ability to neutralize DPPH free radical. The scavenging activity was expressed as the percentage of the radical reduction, providing insight into the efficacy of the samples in eliminating harmful free radicals. BHT was used as a reference compound. The control group consists of an equal volume of ethanol instead of the test compounds. Each experiment was performed in triplicate. The percentage of inhibition was calculated using the following equation: %inhibition = [(A_control_ − A_sample_)/A_control_)] × 100, where A_control_ is the absorbance of the negative control (vehicle only) and A_sample_ is the absorbance of the tested compound. The half-maximal inhibitory concentration (IC_50_) values were determined from the inhibition percentage curves using a nonlinear regression model for each sample, and the results were expressed as mean IC_50_ ± standard deviation (SD).

#### 5.4.2. ABTS Free Radical-Scavenging Activity

Antioxidant activity of the plant extracts and compounds was measured using ABTS free radical-scavenging assay [57]. ABTS•^+^ is a highly coloured radical cation that is produced by reacting a 7mM ABTS•^+^ aqueous solution with 2.45 mM potassium persulfate. The resulting mixture is left at room temperature in the dark for 12 to 16 h to produce the intensely coloured ABTS•^+^ radical cation. This solution is then diluted with ethanol to get the working solution. Samples at varying concentrations were mixed with the ABTS working solution to a final volume ratio of 1:10 (*v*/*v*). Final concentrations were 10, 25, 50, 100, 250, and 500 µg/mL for the extracts and 1, 2.5, 5, 10, 25, 50, and 100 µg/mL for the compounds. The absorbance is measured spectrophotometrically after 6 min of incubation at 734 nm using the SpectraMax^®^ 190 Microplate Reader. Trolox was used as a reference compound. The control group consists of an equal volume of ethanol instead of the test compounds. Each experiment was performed in triplicate. The percentage of inhibition was calculated using the following equation: %inhibition = (A_control_ − A_sample_)/A_control_) × 100, where A_control_ is the absorbance of the negative control (vehicle only) and A_sample_ is the absorbance of the tested compound. The IC_50_ values were determined from the inhibition percentage curves using a nonlinear regression model for each sample, and the results were expressed as mean IC_50_ ± SD.

### 5.5. Statistical Analysis

Each experiment was run in triplicate. Results were expressed as mean ± SD. IC_50_ values were calculated using GraphPad Prism software version 9.0. The data was analyzed statistically using IBM SPSS Statistics version 25.0 (IBM Corp., Armonk, NY, USA). Normality was assessed using the Shapiro–Wilk test, and homogeneity of variances was evaluated using the Levene test. Differences between groups that exhibited a normal distribution and homogeneity of variances were examined using one-way ANOVA followed by a post hoc Tukey test; if variances were not homogeneous, a post hoc Dunnett T3 test was used. For differences between groups that did not exhibit a normal distribution, the Kruskal–Wallis test was used. *p*-values less than 0.05 were reported as statistically significant.

## Figures and Tables

**Table 1:** ^1^H NMR of the isolated compounds.

H	12	13	14	15	16
**1**	-	-	-	-	-
**2**	-	-	-	-	-
**3**	5.8 (d, *J* = 9.6)	5.9 (d, *J* = 9.6)	6.1 (d, *J* = 9.6)	5.8 (d, *J* = 9.0)	6.2 (d, *J* = 9.2)
**4**	7.6 (d, *J* = 9.2)	7.8 (d, *J* = 9.6)	7.8 (d, *J* = 9.6)	7.7 (d, *J* = 9.0)	7.8 (d, *J* = 9.6)
**5**	7.1 (s)	7.2 (s)	7.3 (s)	7.1 (s)	7.2 (s)
**6**	-	-	-	-	-
**7**	-	-	-	-	-
**8**	6.4 (s)	6.5 (s)	6.7 (s)	6.4 (s)	6.8 (s)
**9**	-	-	-	-	-
**10**	-	-	-	-	-
**1′**	4.6 (d, *J* = 7.6)	4.7 (d, *J* = 8.4)	4.6 (d, *J* = 7.8)	4.7 (d, *J* = 7.8)	4.4 (d, *J* = 7.6)
**2′**	3.4 (m)	3.5 (m)	3.2 (m)	3.7 (m)	3.5 (m)
**3′**	3.3 (m)	3.4 (m)	3.2 (m)	3.6 (m)	3.5 (m)
**4′**	3.2 (m)	3.3 (m)	3.0 (m)	3.4 (m)	3.5 (m)
**5′**	3.3 (m)	3.6 (m)	3.5 (m)	3.3 (m)	3.2 (m)
**6′**	3.6 (dd, *J* = 6.0, 12.8) 3.8 (dd, *J* = 2.4, 12.0)	3.6 (m) 4.0 (m)	3.5 (m) 3.9 (m)	3.7 (m) 3.9 (m)	3.8(dd, *J* = 6.0, 11.2) 4.1 (d, *J* = 12.0)
**1″**	-	5.0 (d, *J* = 2.4)	4.1 (d, *J* = 7.8)	4.6 (d, *J* = 7.2)	5.0 (d, *J* = 6.4)
**2″**	-	3.9 (d, *J* = 2.4)	2.9 (m)	3.2 (m)	3.2 (m)
**3″**	-	-	2.9 (m)	3.3 (m)	3.7 (m)
**4″**	-	3.7 (d, *J* = 9.0) 3.9 (d, *J* = 9.0)	3.2 (m)	3.2 (m)	3.2 (m)
**5″**	-	3.5 (s)	2.8 (m) 3.6 (m)	3.4 (m)	3.2 (m)
**6″**	-	-	-	3.6 (m) 3.8 (m)	3.6 (m) 3.7 (m)

**Table 2:** ^13^C-NMR of compound **12**–**16**.

C	12	13	14	15	16
**1**	-	-	-	-	-
**2**	164.0	165.5	161.0	165.9	164.7
**3**	106.4	107.6	112.1	106.2	111.9
**4**	145.5	147.1	145.2	147.1	145.8
**5**	113.6	114.9	114.7	115.2	114.5
**6**	146.5	148.0	142.7	148.9	142.2
**7**	162.0	154.5	152.2	155.1	150.5
**8**	104.0	105.3	103.6	105.8	103.8
**9**	153.0	154.5	152.0	155.1	150.5
**10**	107.4	108.8	110.9	105.5	112.1
**1′**	103.0	104.3	102.8	104.4	101.0
**2′**	73.4	74.3	73.6	74.3	72.7
**3′**	77.0	77.5	76.1	86.9	75.5
**4′**	70.2	71.7	70.1	70.1	69.1
**5′**	76.1	77.2	76.1	78.1	75.7
**6′**	61.3	68.8	68.4	62.9	68.1
**1″**	-	110.9	104.3	105.3	102.5
**2″**	-	78.0	73.6	75.7	73.1
**3″**	-	80.5	76.8	77.8	75.3
**4″**	-	74.9	70.0	71.5	69.5
**5″**	-	65.4	66.1	78.0	75.8
**6″**	-	-	-	62.6	60.6

**Table 3:** Inhibitory effects of methanolic extract and phases on ABTS and DPPH free radicals.

Plant Extract	IC_50_ (µg/mL)	
	ABTS	DPPH
SM	33.5 ± 1.2	317.6 ± 10.0 *^,b^
SMPE	268.7 ± 15.9 *^,a^	>500
SMC	18.7 ± 0.2	219.1 ± 8.9 *^,b^
SME	43.6 ± 1.1 *^,a^	12.6 ± 0.8
SMW	37.7 ± 3.7 *^,a^	262.2 ± 20.8 *^,b^
Trolox	4.9 ± 0.0	-
BHT	-	7.3 ± 0.4

Data are expressed as mean ± SD (*n* = 3). ^a^: Statistical significance was determined by Kruskal–Wallis. ^b^: Statistical significance was determined by one-way analysis of variance (ANOVA) followed by post hoc Tukey test. (*): *p* < 0.05 indicates a significant difference compared to the reference compound. SM: *P. szowitsii* total extract; SMPE: *P. szowitsii* petroleum ether layer; SMC: *P. szowitsii* chloroform layer; SME: *P. szowitsii* ethylacetate layer; SMW: *P. szowitsii* water layer.

**Table 4:** Antioxidant activities of SME fractions on ABTS and DPPH free radicals.

Fractions	IC_50_ (µg/mL)	
	ABTS	DPPH
SME-1	117.8 ± 1.4 *	201.7 ± 2.6 *
SME-2	16.8 ± 1.2 *	11.4 ± 0.5 *
SME-3	27.7 ± 1.2 *	17.2 ± 0.6 *
SME-4	24.4 ± 1.2 *	19.8 ± 1.0 *
SME-5	28.7 ± 1.8 *	43.5 ± 0.4 *
SME-6	24.7 ± 1.0 *	38.1 ± 1.5 *
SME-7	50.0 ± 1.6 *	79.3 ± 1.7 *
SME-8	38.1 ± 1.0 *	63.4 ± 3.6 *
SME-9	177.5 ± 2.4 *	160.6 ± 0.6 *
SME-10	120.8 ± 3.6 *	210.2 ± 4.2 *
SME-11	64.8 ± 1.7 *	151.6 ± 8.4 *
SME-12	189.3 ± 6.3 *	>500
Trolox	5.0 ± 0.0	-
BHT	-	7.4 ± 0.0

Data are expressed as mean ± SD (*n* = 3). Statistical significance was determined by one-way ANOVA followed by post hoc Dunnett T3 and Tukey tests. (*): *p* < 0.05 indicates a significant difference compared to the reference compound.

**Table 5:** Antioxidant activities of SMW fractions on ABTS and DPPH free radicals.

Plant Extract	IC_50_ (µg/mL)	
	ABTS	DPPH
SMW-A	123.5 ± 3.5 *	86.9 ± 0.6 *
SMW-B	160.1 ± 7.8 *	101.6 ± 0.5 *
SMW-C	444.9 ± 1.1 *	205.6 ± 0.3 *
SMW-D	64.5 ± 5.7 *	306.9 ± 2.1 *
SMW-E	198.6 ± 5.3 *	135.1 ± 3.3 *
SMW-F	177.6 ± 3.3 *	114.8 ± 4.0 *
SMW-G	>500	>500
SMW-H	>500	>500
Trolox	5.0 ± 0.0	-
BHT	-	8.0 ± 0.1

Data are expressed as mean ± SD (*n* = 3). Statistical significance was determined by one-way ANOVA followed by post hoc Tukey test. (*): *p* < 0.05 indicates a significant difference compared to the reference compound.

**Table 6:** Antioxidant activities of compounds obtained SME and SMW.

Compound	IC_50_ (µg/mL)	
	ABTS	DPPH
Protocatechuic acid	15.3 ± 0.0 *^,a^	10.2 ± 0.3 *^,b^
Caffeic acid	3.6 ± 0.3	2.7 ± 0.2 *^,b^
Chlorogenic acid	5.3 ± 0.4	4.8 ± 0.0 *^,b^
Chlorogenic acid methyl ester	8.5 ± 0.0	8.4 ± 0.6
1,5-*O*-dicaffeoyl quinic acid	6.6 ± 0.0	5.5 ± 0.1 *^,b^
3,5-*O*-dicaffeoyl quinic acid	6.7 ± 0.2	3.4 ± 0.0 *^,b^
1,5-*O*-dicaffeoyl quinic acid methyl ester	4.1 ± 0.0	2.3 ± 0.2 *^,b^
4,5-*O*-dicaffeoyl quinic acid methyl ester	5.0 ± 0.1	2.8 ± 0.0 *^,b^
Scopolin	36.1 ± 5.4 *^,a^	>100
Esculetin 6-*O*-*β*-glucopyranoside	67.6 ± 4.36 *^,a^	>100
Esculetin 6-*O*-*β*-apiofuranosyl-(1→6)-*β*-glucopyranoside	>100	>100
Esculetin 6-O-*β*-glucopyranosyl-(1→3)-*β*-glucopyranoside	>100	>100
Esculetin 6-*O*-*β*-xylosyl-(1→6)-*β*-glucopyranoside	>100	>100
Esculetin 6-O-*β*-glucopyranosyl-(1→6)-*β*-glucopyranoside	>100	>100
2,4,6 trimethoxyphenyl-1-*O*-*β*-glucopyranoside	>100	>100
2,4,6 trimethoxyphenyl-1-*O*-*β*-apiofuranosyl-(1→6)-*β*-glucopyranoside	16.7 ± 0.8	>100
Trolox	4.8 ± 0.1	-
BHT	-	7.6 ± 0.0

Data are expressed as mean ± SD (*n* = 3). ^a^: Statistical significance was determined by one-way analysis of variance (ANOVA) followed by post hoc Tukey test. ^b^: Statistical significance was determined by one-way ANOVA followed by post hoc Dunnett T3 test. (*): *p* < 0.05 indicates a significant difference compared to the reference compound.

## Data Availability

The original contributions presented in this study are included in the article/Supplementary Material. Further inquiries can be directed to the corresponding author.

## Supplementary Materials

The following supporting information can be downloaded at: https://www.mdpi.com/article/10.3390/molecules31091507/s1; Figures S1–S25. NMR (^1^H, ^13^C, HMBC, HSQC, COSY) and MS spectra of compounds **14**–**16**, Figures S26–S31. Antioxidant activity calibration curves.
