# Supplementary material for: Bioactive Phenolic Acid Derivatives and Undescribed Esculetin Glycosides from Pseudopodospermum szowitzii (DC.) Kuth
Source: Molecules. 2026 May 1;31(9):1507. doi: 10.3390/molecules31091507 (PMC13165379; doi:10.3390/molecules31091507)
Supplement: Supplementary file 1 [file molecules-31-01507-s001.zip › molecules-4232309-supplementary.pdf]

Bioactive Phenolic Acid Derivatives and Undescribed Esculetin Glycosides from *Pseudopodospermum szowitzii* (DC.) Kuth.

Sezen Yılmaz Sarıaltın, Özlem Bahadır Acıkara, Büşra Yaylacı and Christian Zidorn

Compounds **10** and **11** were identified based on their MS and NMR values and comparison with literature data. ^1H and ^{13}C-NMR spectra of compound **10** indicated that compound **10** contains a tetrasubstituted symmetrical benzene cycle with three methoxyl groups, of which two are equivalent and one hydroxyl group. *β*-Glucopyranose as the sugar moiety was identified from NMR spectra, and the linkage position of the sugar unit at C-1 of the aglycone was proved by an HMBC correlation between the signal of the anomeric proton δ 4.8 ppm (1H, d, *J* = 7.6 Hz, H-1') and the signal assignable to the carbon in position C-1 of the aglycone at δ 154.7 ppm. ^1H and ^{13}C-NMR spectra of compound **11** were similar to those of compound **10**. However, the downfield-shifted C-6′ carbon of glucose (δ_C 67.3) suggested that another monosaccharide, which had already been identified as apiofuranoside from the signal observed in ^{13}C and ^1H NMR spectra, was connected via the O-6′ position of glucose. The structure of compound **11** was established as 2,4,6-trimethoxyphenyl-1-*O*-*β*-apiofuranosyl-(1→6)-*β*-glucopyranoside, and the structure was confirmed by comparison of literature data [11-12].

Compound **12** was obtained as a yellow oil residue, and the compound's molecular weight was determined as *m*/*z* = 339.91 [M-H]$^-$. By comparison of the ^1H and ^{13}C NMR data, the structure of compound **12** was considered as a coumarin derivative. The existence of the coumarin skeleton was confirmed by the observed signals at δ 5.8 (1H, d, *J* = 9.6 Hz, H-3), δ 7.6 (1H, d, *J* = 9.2 Hz, H-4), and δ 106.4 (C-3), δ 145.5 (C-4), which were very typical for a benzo-*α* pyron skeleton. Further, two singlet proton signals, at δ 6.4 and δ 7.1 ppm, indicated two substituents at C-6 and C-7 of the simple coumarin ring. The sugar moiety was established by the presence of an anomeric proton signal δ 4.6 (d, *J*=7.6 Hz) and an anomeric carbon signal δ 103.0, and both ^{13}C as well as ^1H NMR values observed from spectra supported the presence of glucose in *β*-configuration as the sugar moiety. HMBC correlation between δ 4.6 (d, *J*=7.6 Hz) ppm proton signal and δ 146.3 ppm carbon signal displayed that the glucose molecule was attached to the aglycone from the C-6 position. As a result, the structure of compound **12** was established as 6,7 dihydroxy coumarin-6-*O*-*β*- glucopyranoside, in other words, esculin [13-14].

Chemical shifts in the ^1H- and ^{13}C-NMR spectra (Tables 1 and 2) of **13** revealed that the compound was also an esculetin derivative as compared with compound **12**, which was established as esculetin-6-*O*-*β*-glucopyranoside. From the ^{13}C-NMR spectrum, 20 carbon signals were observed, nine of which were similar to those of the 6,7-dihydroxy coumarin structure. The remaining 11 carbon signals indicated two different sugar moieties by the presence of two different anomeric proton and carbon signals at δ 4.7 (d, *J* = 7.8 Hz, H-1'), δ 5.0 (d, *J* = 2.4 Hz, H-1″), and δ 104.3 (C-1'), δ 110.9 (C-1″). All data obtained from ^1H- and ^{13}C-NMR spectra indicated that the compound contains apiose and glucose as sugar moieties. The downfield-shifted C-6′ carbon (δ_C 68.8) and H-6' (δ_H 4.0, 3.5) proton of glucose are considered an apiose connection from the C-6′ position of glucose. Cross-peaks between δ 4.0 (H-6'a), δ 3.6 (H-6'b) proton signals and δ 110.9 (C-1″) carbon signals confirmed the connection of apiose to the glucose. This was between the glucosyl anomeric proton at δ 4.7 (d, *J*=7.8 Hz, H-1') and the carbon signal δ 148.0 (C-6), indicating that the apiosyl-(1→6)-glucosyl moiety was attached to the C-6 hydroxyl group. The molecular weight of compound **13** was determined from the MS spectrum as m/z 471.20 [M-H]$^-$. Thus, the structure of **13** was determined to be esculetin-6-*O*-*β*-D-apiofuranosyl-(1→6)-*O*-*β*-glucopyranoside, and the spectral data were confirmed by comparison with the literature [13, 15].

Display Report

Analysis Info

Analysis Name	D:\Data\HRMS\Ozlem_Acikara\260224\SM3-D-neg.d	Acquisition Date	2/27/2024 9:45:30 AM
Method	DEFAULT.m	Operator	Demo User
Sample Name	SM3-D-neg	Instrument	maXis II ETD 1823391.22368
Comment			

Acquisition Parameter

Source Type	ESI	Ion Polarity	Negative	Set Nebulizer	0.3 Bar
Focus	Not active	Set Capillary	2100 V	Set Dry Heater	200 °C
Scan Begin	50 m/z	Set End Plate Offset	-500 V	Set Dry Gas	4.0 l/min
Scan End	1000 m/z	Set Charging Voltage	0 V	Set Divert Valve	Source
		Set Corona	0 nA	Set APCI Heater	0 °C

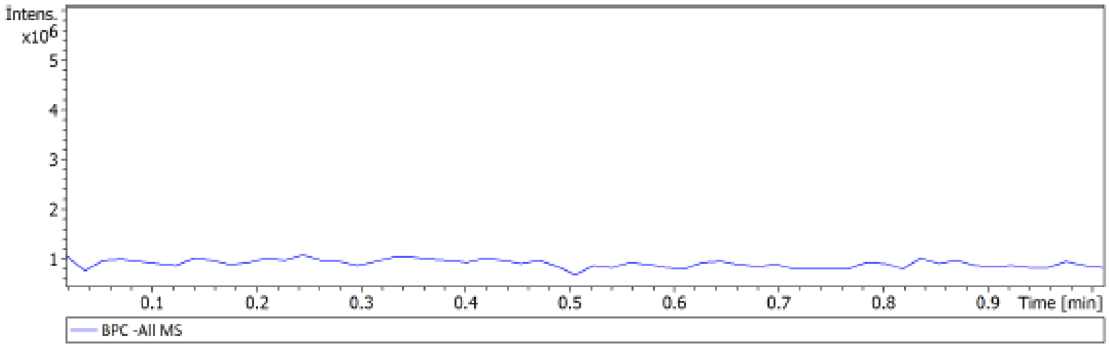

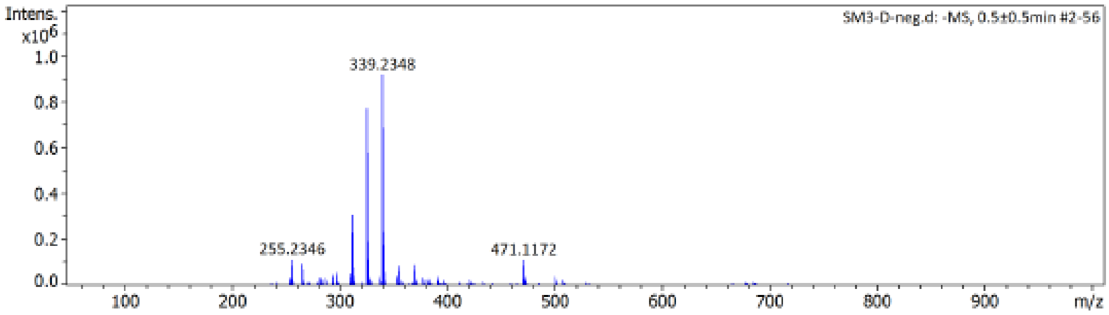

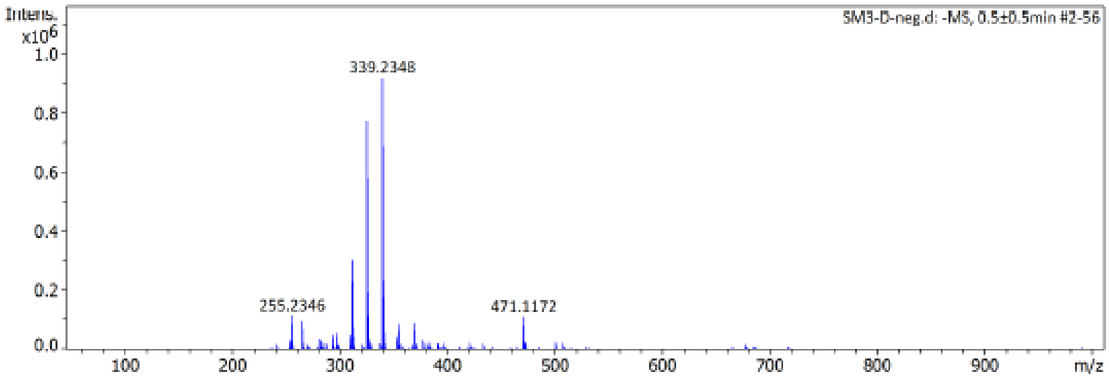

SM3-D-neg.d

Bruker Compass DataAnalysis 5.2 printed: 2/27/2024 9:53:22 AM by: demo Page 1 of 1

Figure S1. HR-MS spectrum of compound **14**.

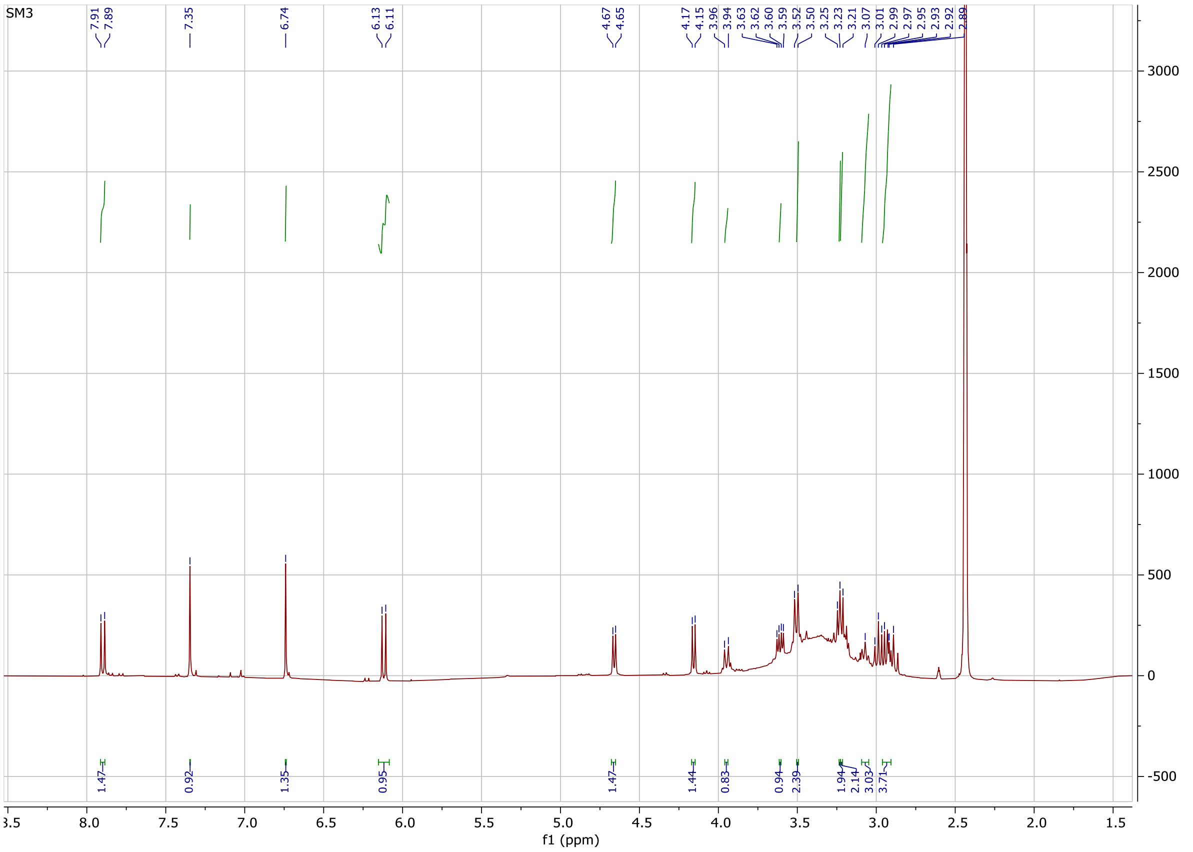

Figure S2. ^{1}H-NMR spectrum of compound **14**.

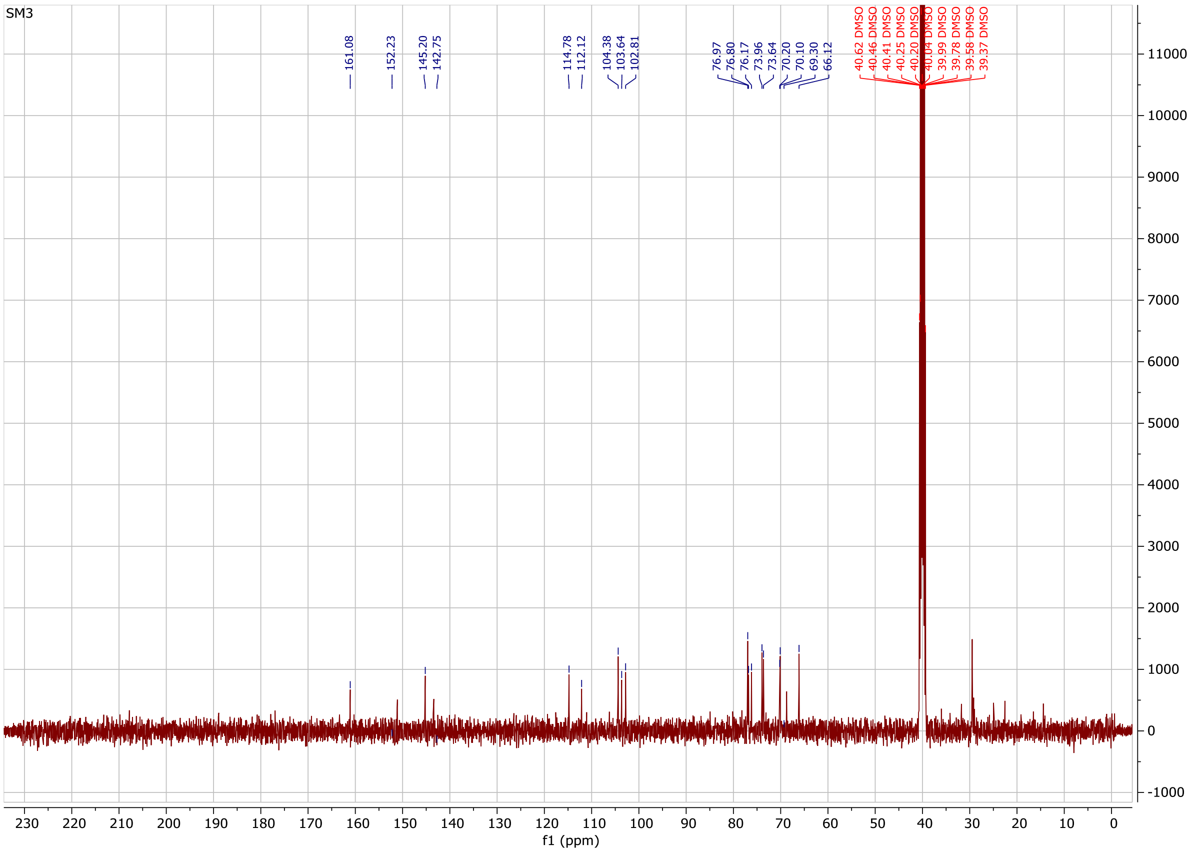

Figure S3. ^{13}C NMR spectrum of compound **14**.

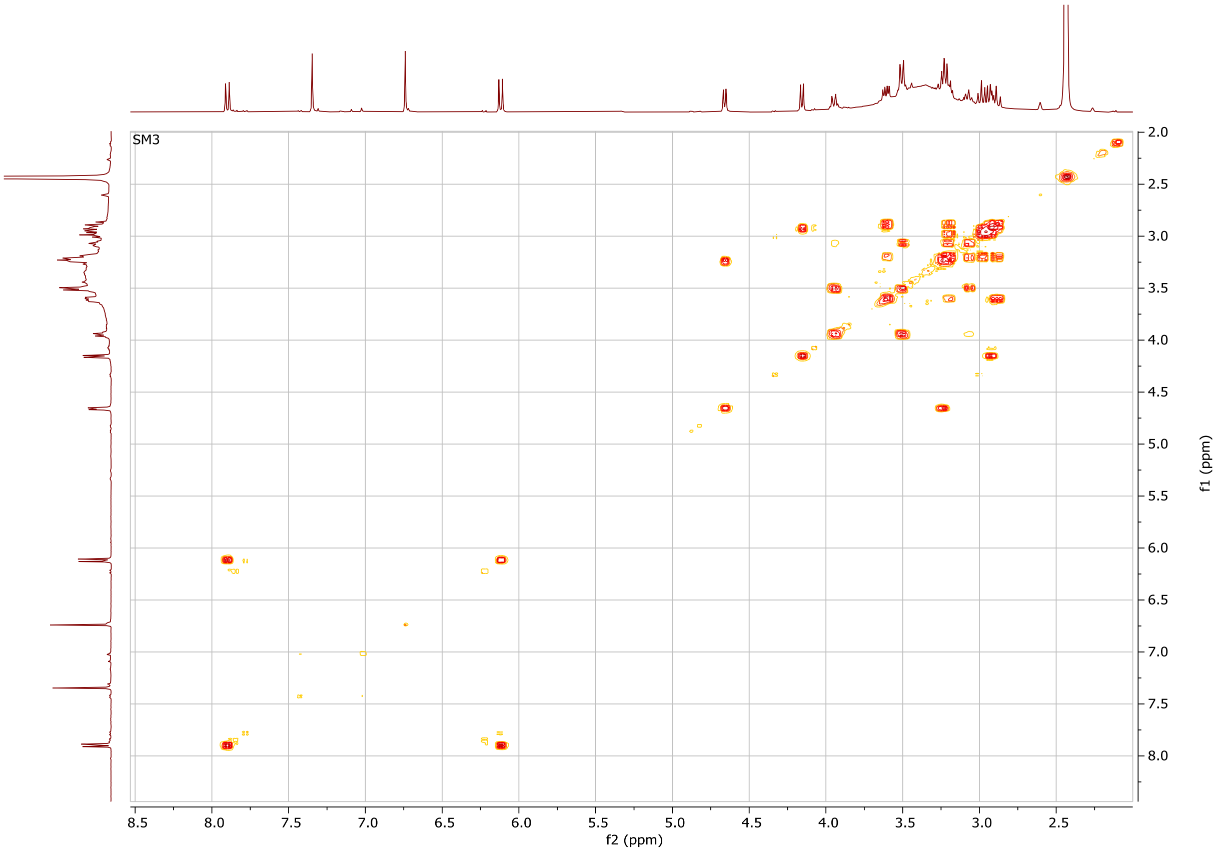

Figure S4. COSY spectrum of compound **14**.

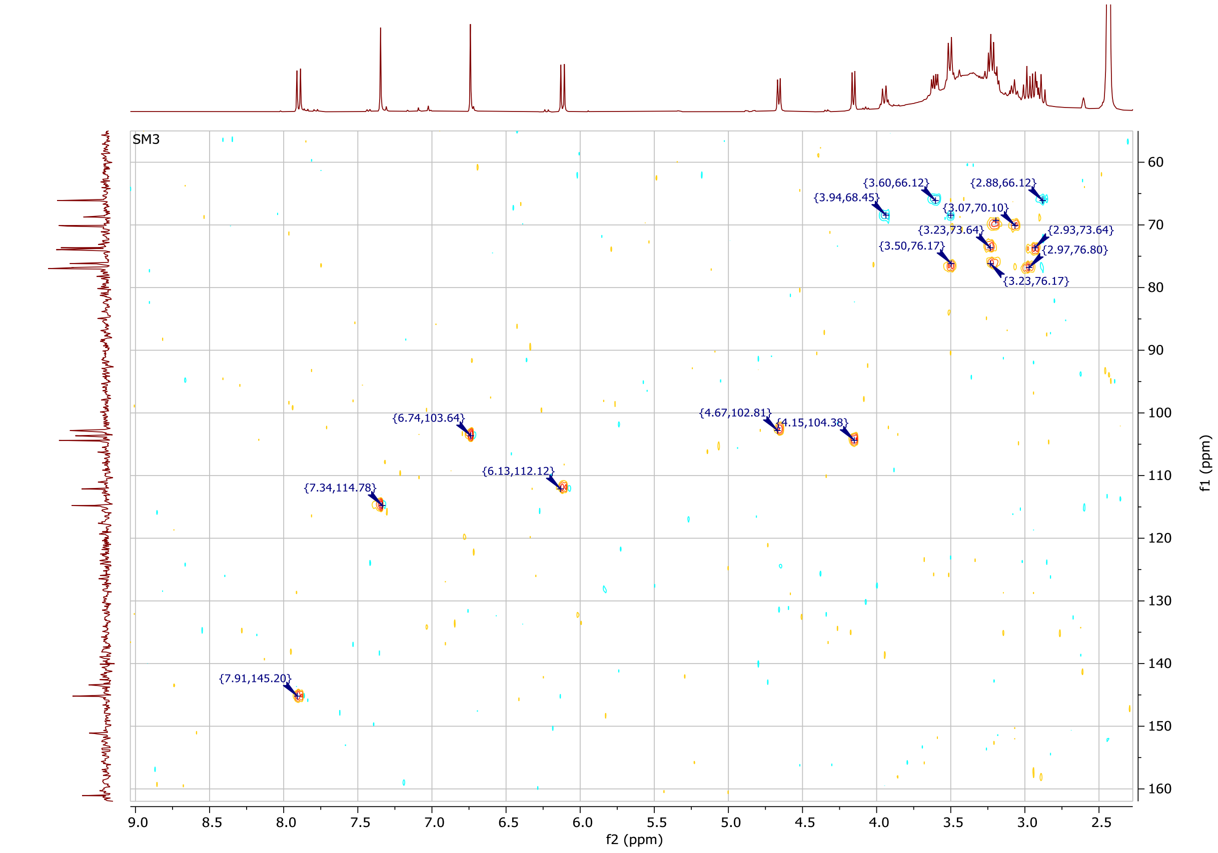

Figure S5. HSQC spectrum of compound **14**.

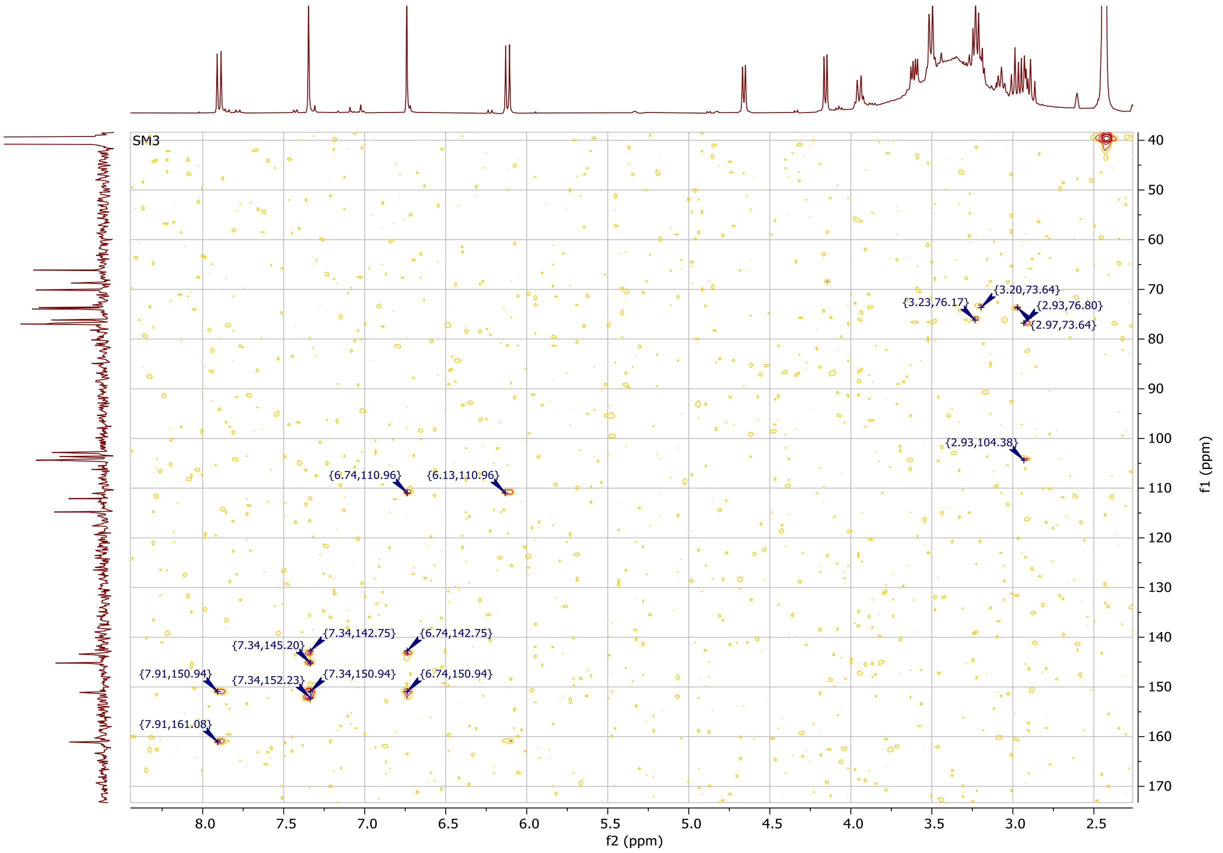

Figure S6. HMBC spectrum of the compound **14**.

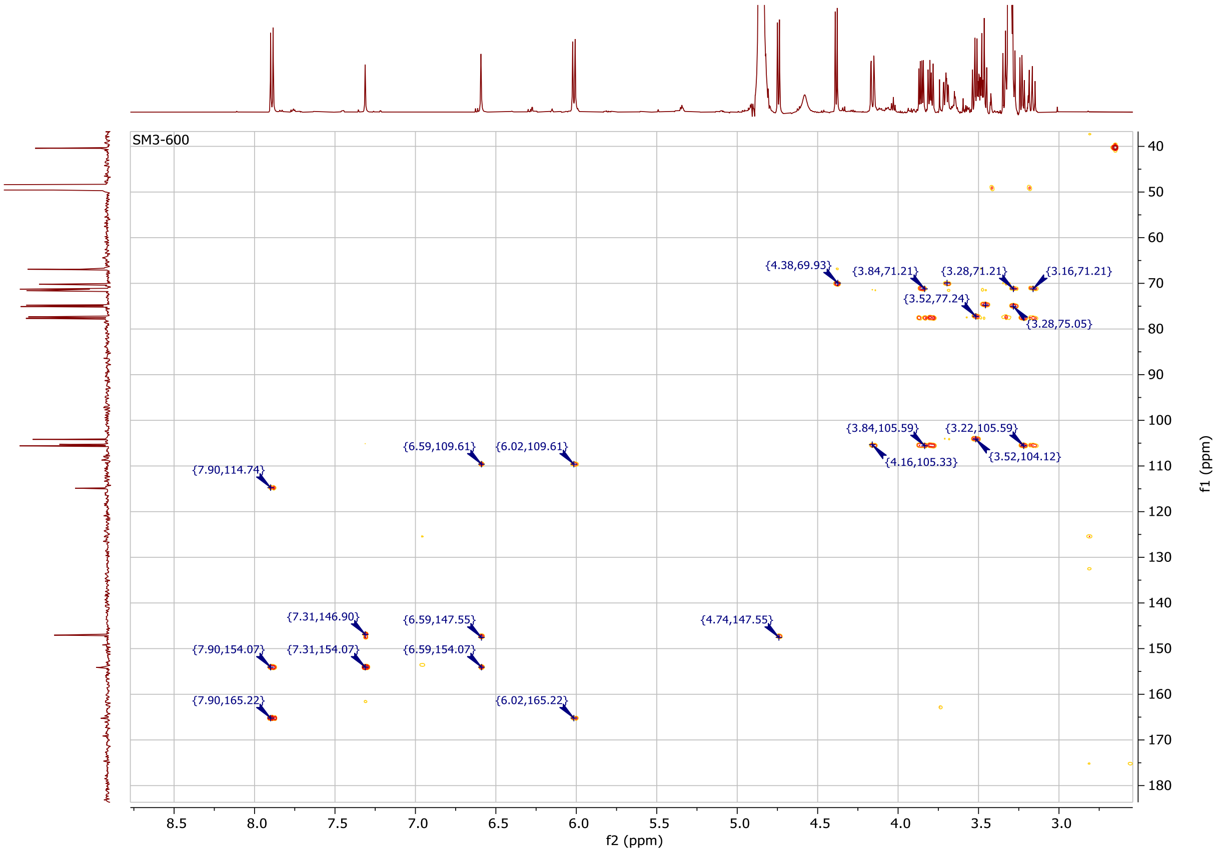

Figure S7. HMBC (600 MHz NMR) spectrum of the compound **14**.

Display Report

Analysis Info

Analysis Name	D:\Data\HRMS\Ozlem_Acikara\260224\SM3-B-neg.d	Acquisition Date	2/27/2024 9:34:31 AM
Method	DEFAULT.m	Operator	Demo User
Sample Name	SM3-B-neg	Instrument	maXis II ETD 1823391.22368
Comment			

Acquisition Parameter

Source Type	ESI	Ion Polarity	Negative	Set Nebulizer	0.3 Bar
Focus	Not active	Set Capillary	2100 V	Set Dry Heater	200 °C
Scan Begin	50 m/z	Set End Plate Offset	-500 V	Set Dry Gas	4.0 l/min
Scan End	1000 m/z	Set Charging Voltage	0 V	Set Divert Valve	Source
		Set Corona	0 nA	Set APCI Heater	0 °C

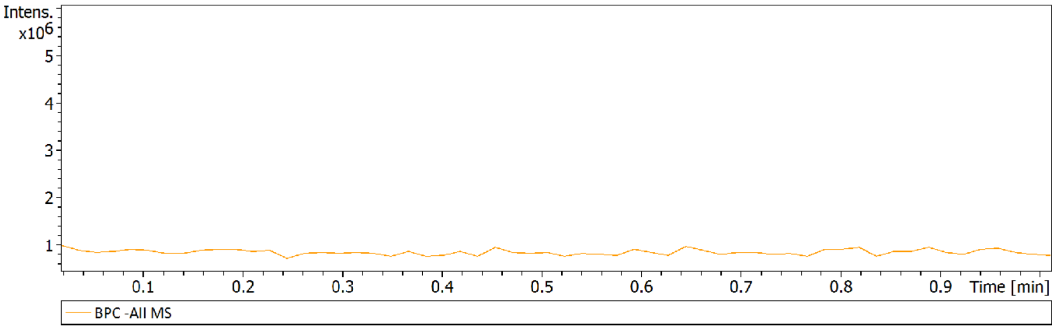

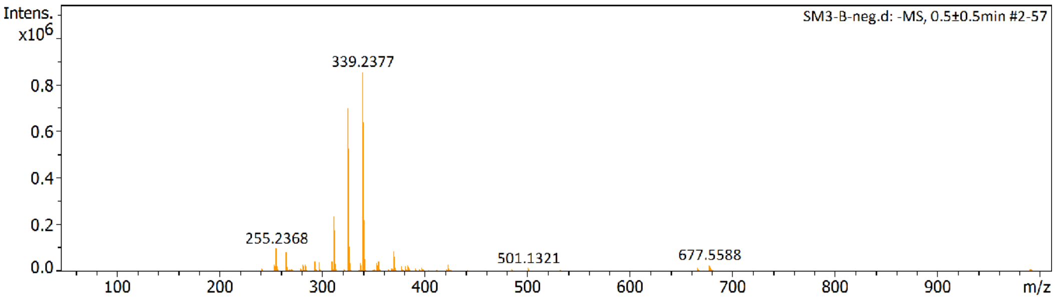

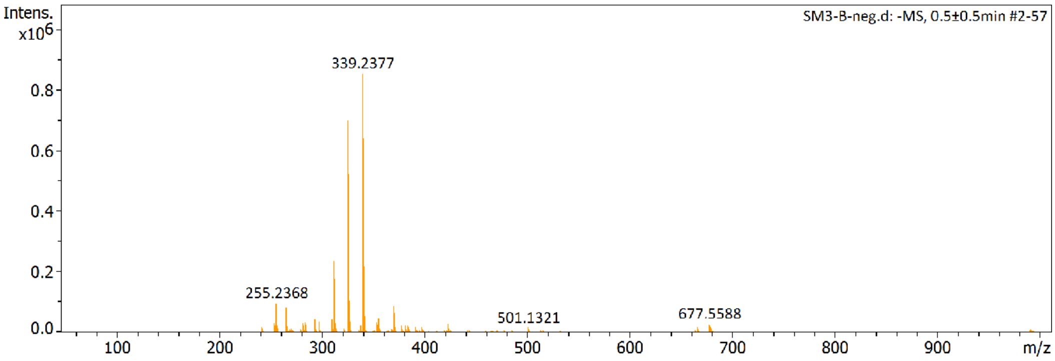

SM3-B-neg.d

Bruker Compass DataAnalysis 5.2 printed: 2/27/2024 9:54:20 AM by:

Figure S8. HR-MS spectrum of compound **15**.

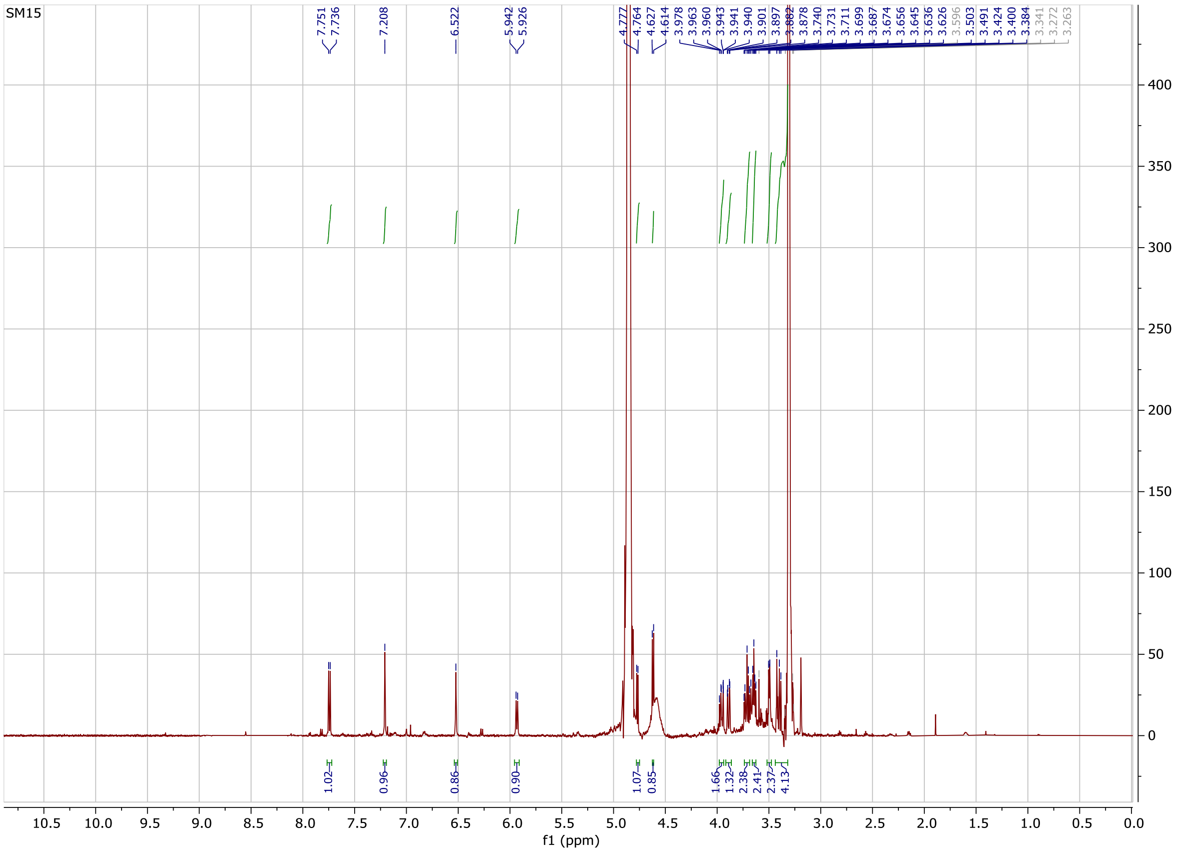

Figure S9. ^{1}H NMR spectrum of compound **15**.

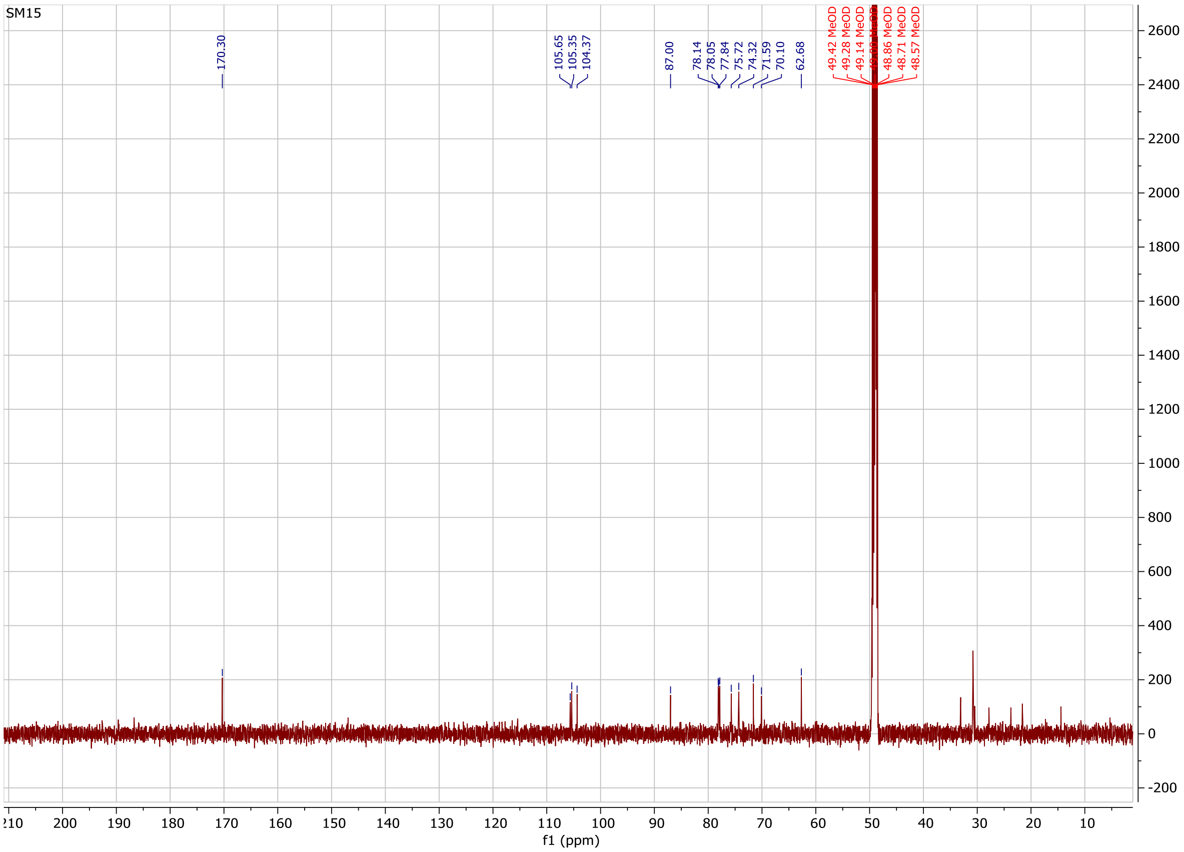

Figure S10. ^{13}C NMR spectrum of compound **15**.

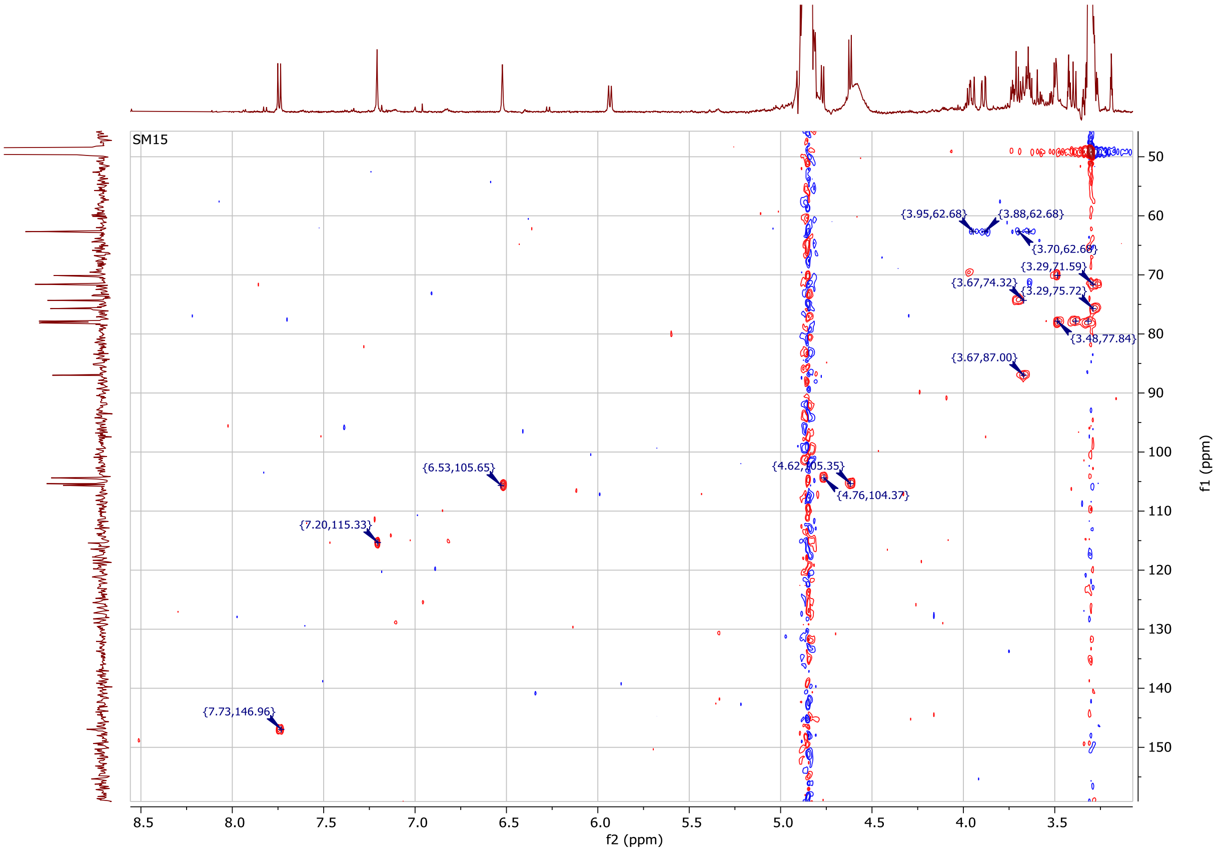

Figure S11. HSQC spectrum of the compound **15**.

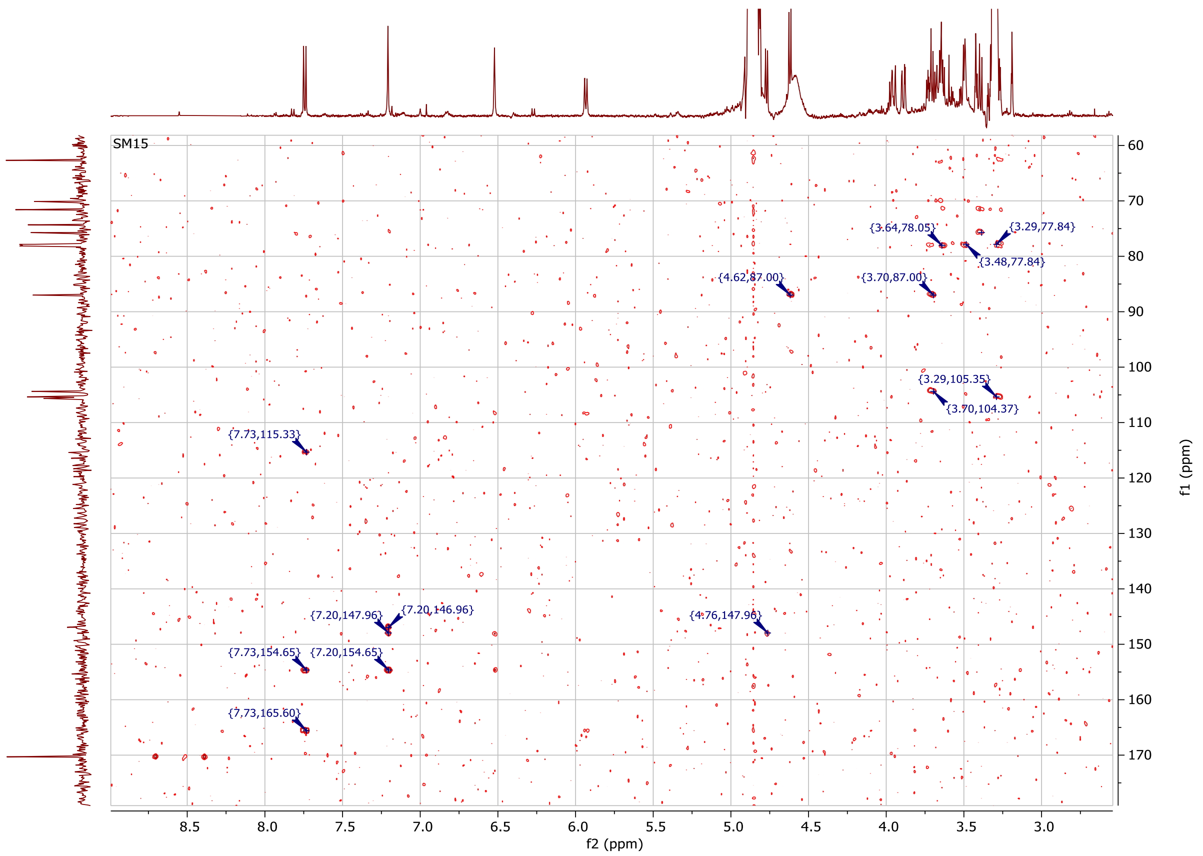

Figure S12. HMBC spectrum of the compound **15**.

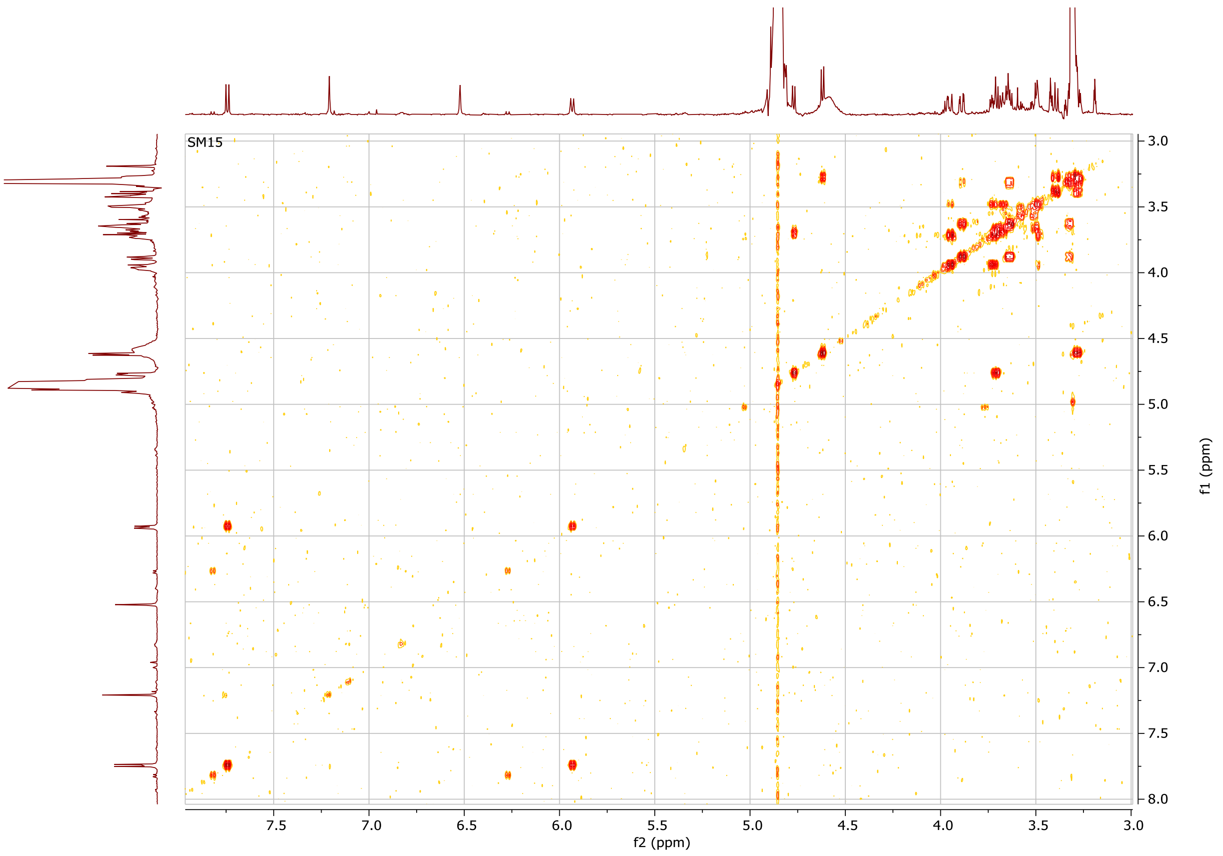

Figure S13. COSY spectrum of the compound **15**.

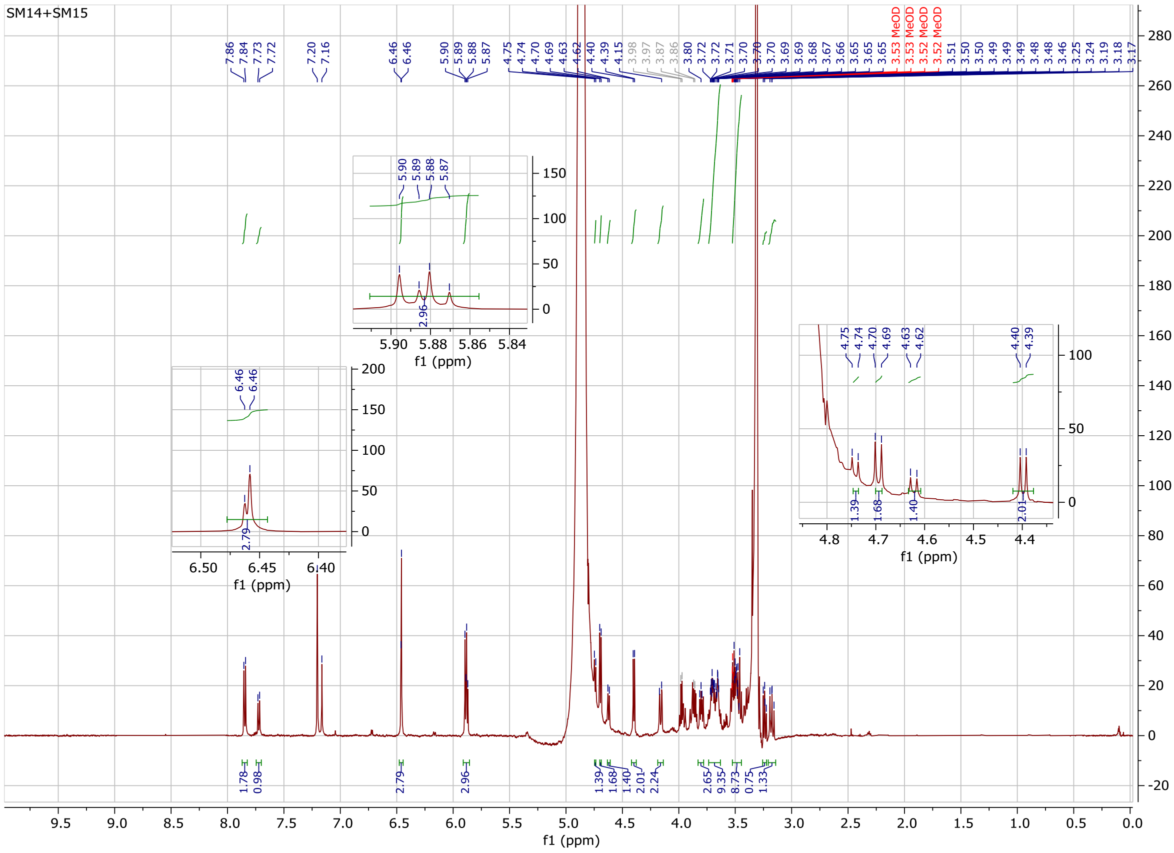

Figure S14. ^{1}H-NMR spectrum of the compound **14** and **15** mixture.

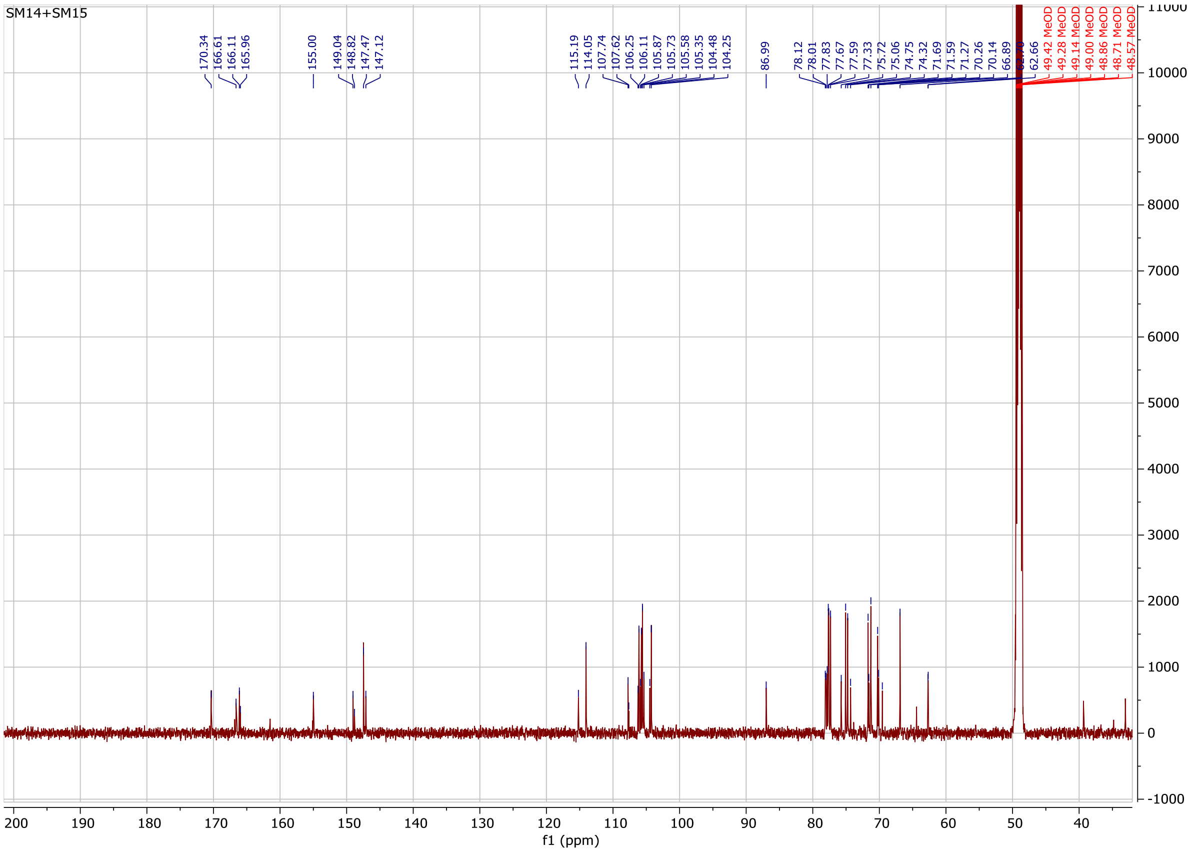

Figure S15. ^{13}C-NMR spectrum of the compound **14** and **15** mixture.

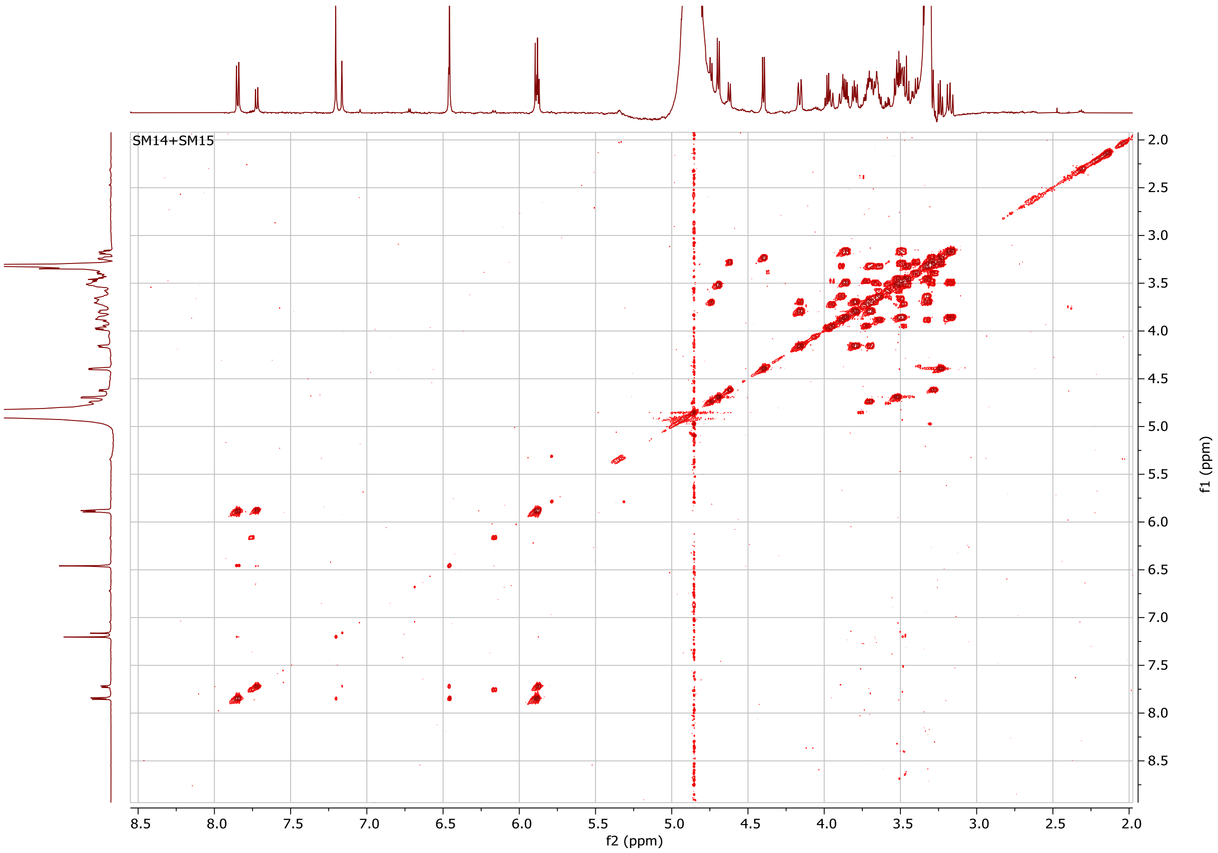

Figure S16. COSY spectrum of the compound **14** and **15** mixture.

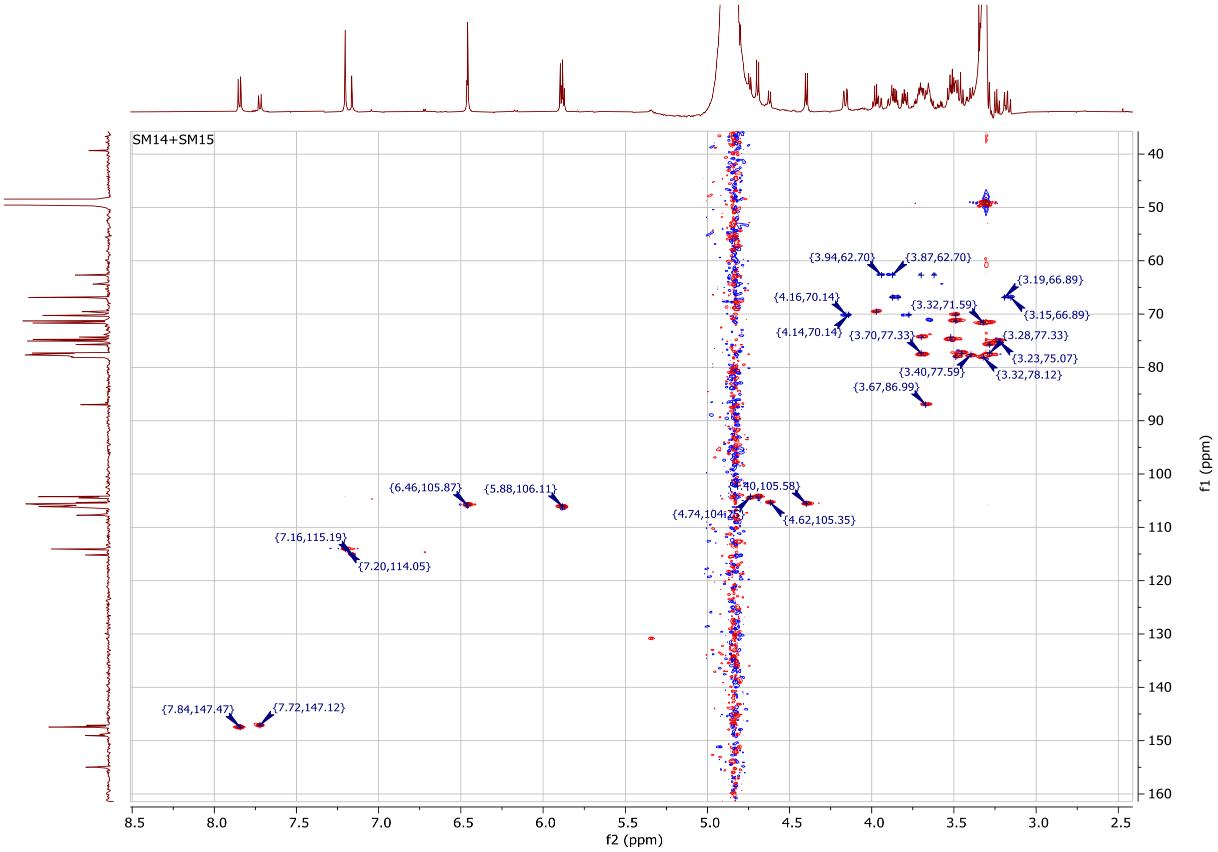

Figure S17. HSQC spectrum of the compound **14** and **15** mixture.

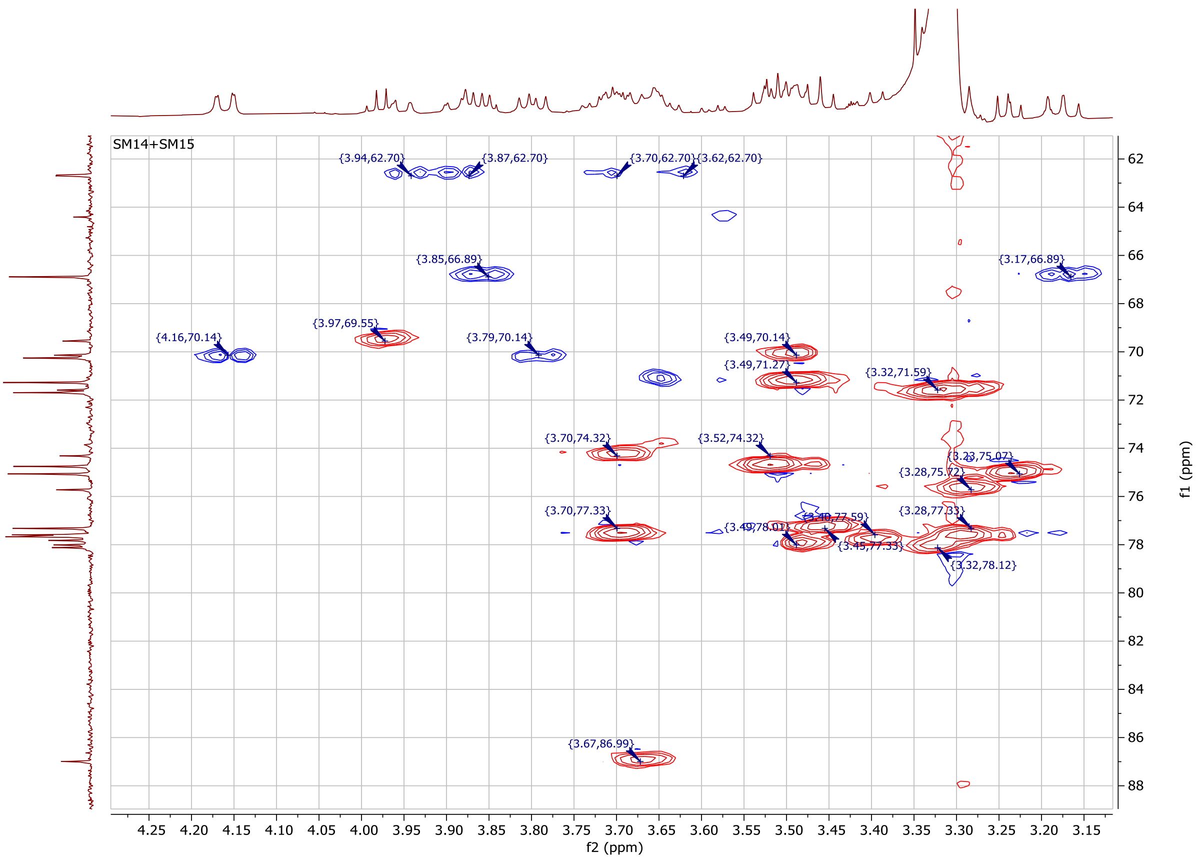

Figure S18. Detailed HSQC spectrum of the compound **14** and **15** mixture.

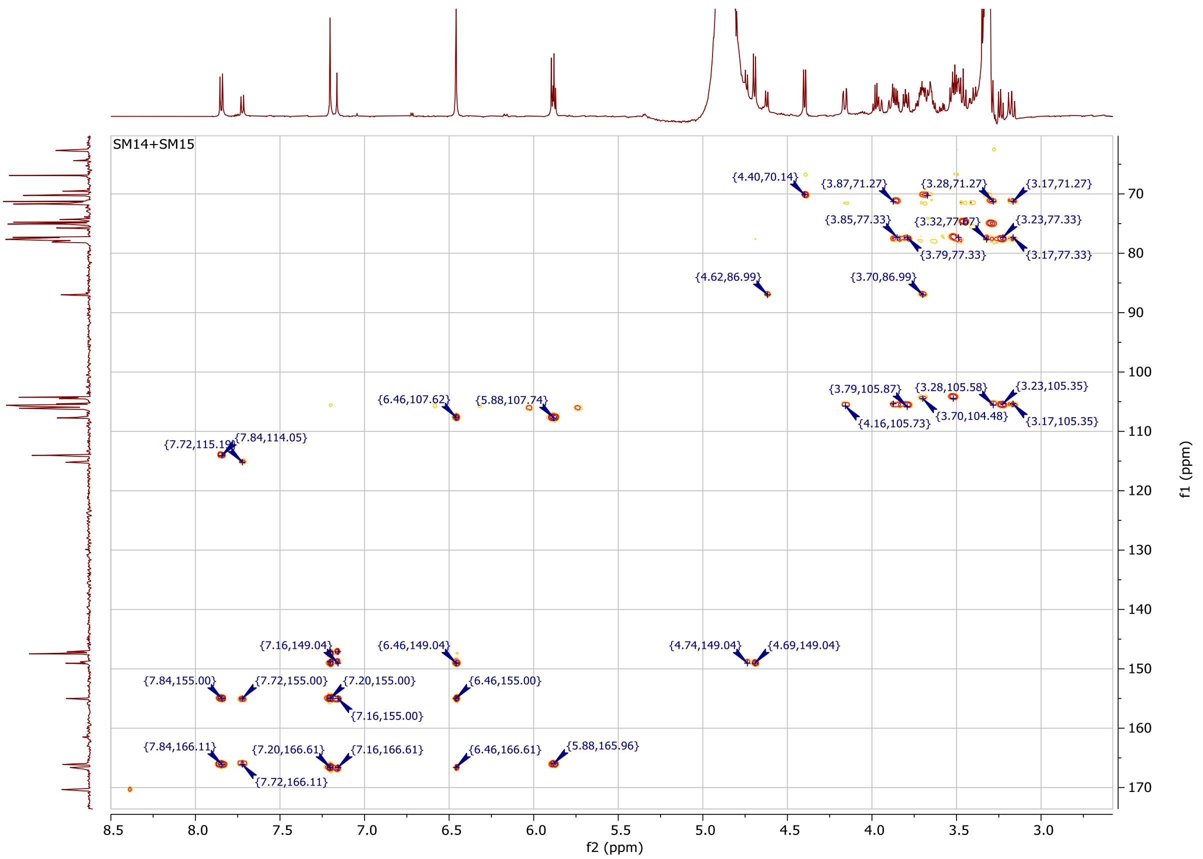

Figure S19. HMBC spectrum of the compound **14** and **15** mixture.

Display Report

Analysis Info

		Acquisition Date	2/26/2024 2:16:23 PM
Analysis Name	D:\Data\HRMS\Ozlem_Acikara\260224\SM4.d		
Method	DEFAULT.m	Operator	Demo User
Sample Name	SM4	Instrument	maXis II ETD 1823391.22368
Comment			

Acquisition Parameter

Source Type	ESI	Ion Polarity	Positive	Set Nebulizer	0.3 Bar
Focus	Not active	Set Capillary	3500 V	Set Dry Heater	200 °C
Scan Begin	50 m/z	Set End Plate Offset	-500 V	Set Dry Gas	4.0 l/min
Scan End	3000 m/z	Set Charging Voltage	0 V	Set Divert Valve	Source
		Set Corona	0 nA	Set APCI Heater	0 °C

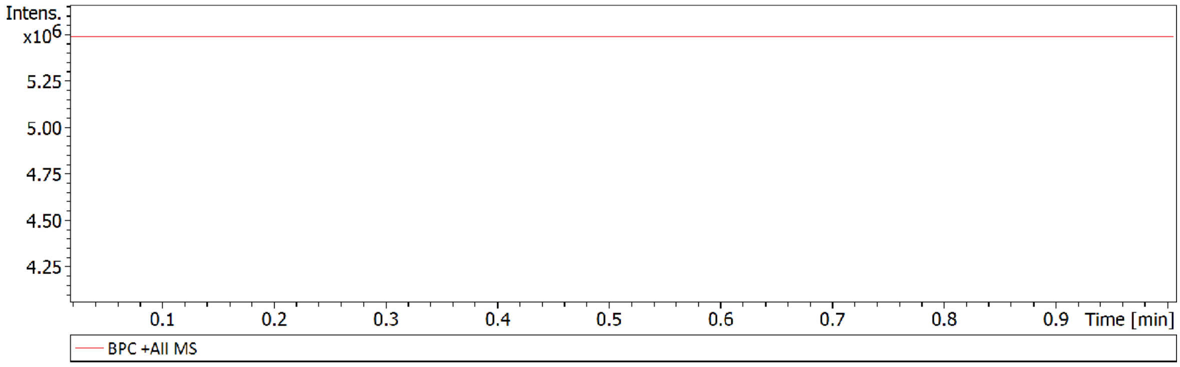

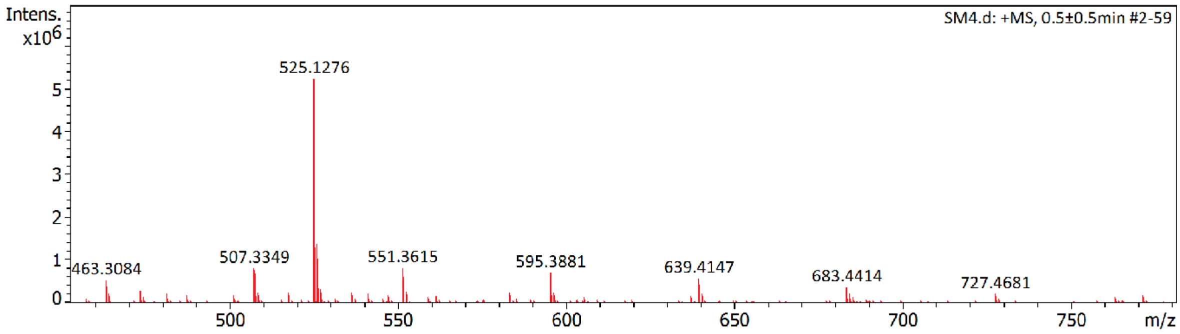

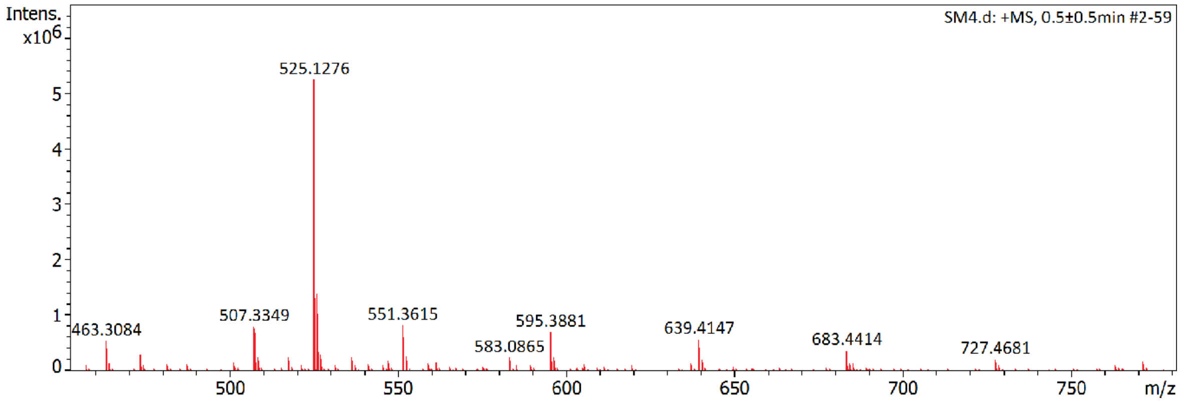

Figure S20. HR-MS spectrum of compound **16**.

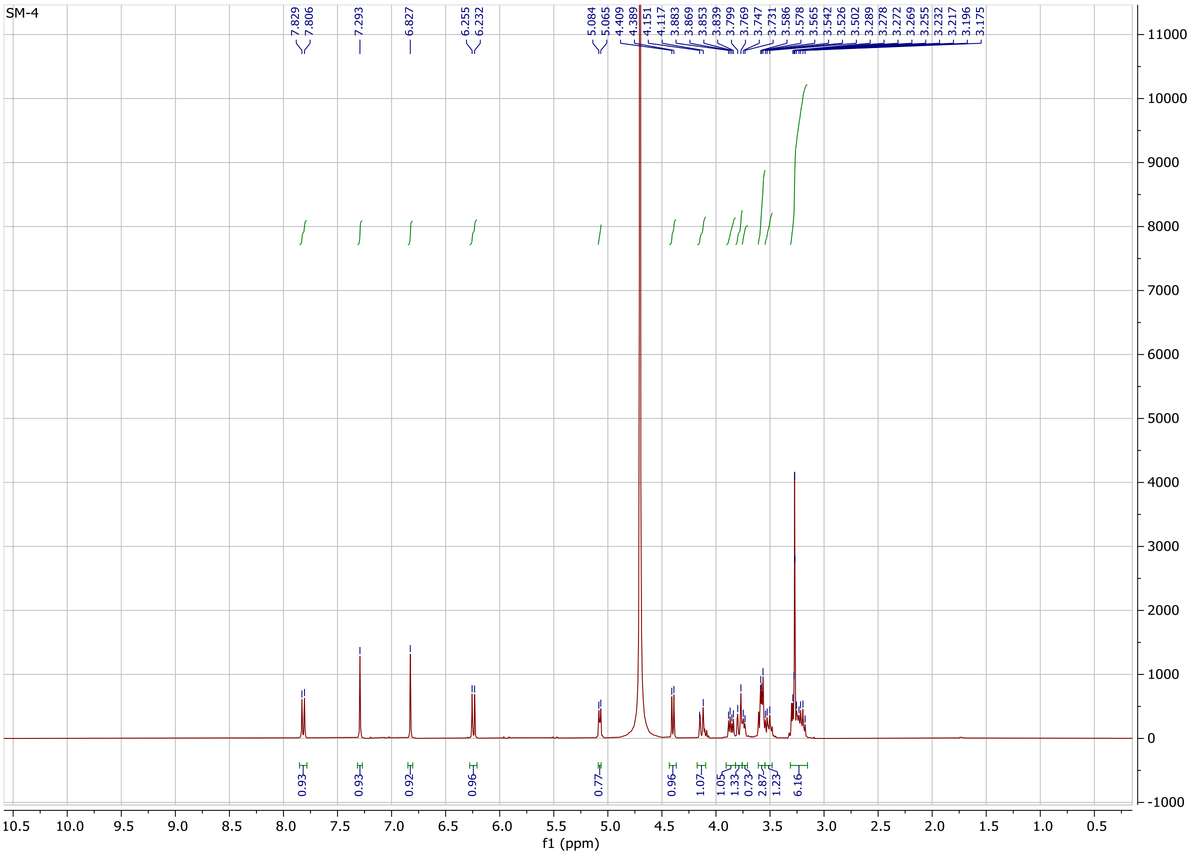

Figure S21. ^{1}H-NMR spectrum of compound **16**.

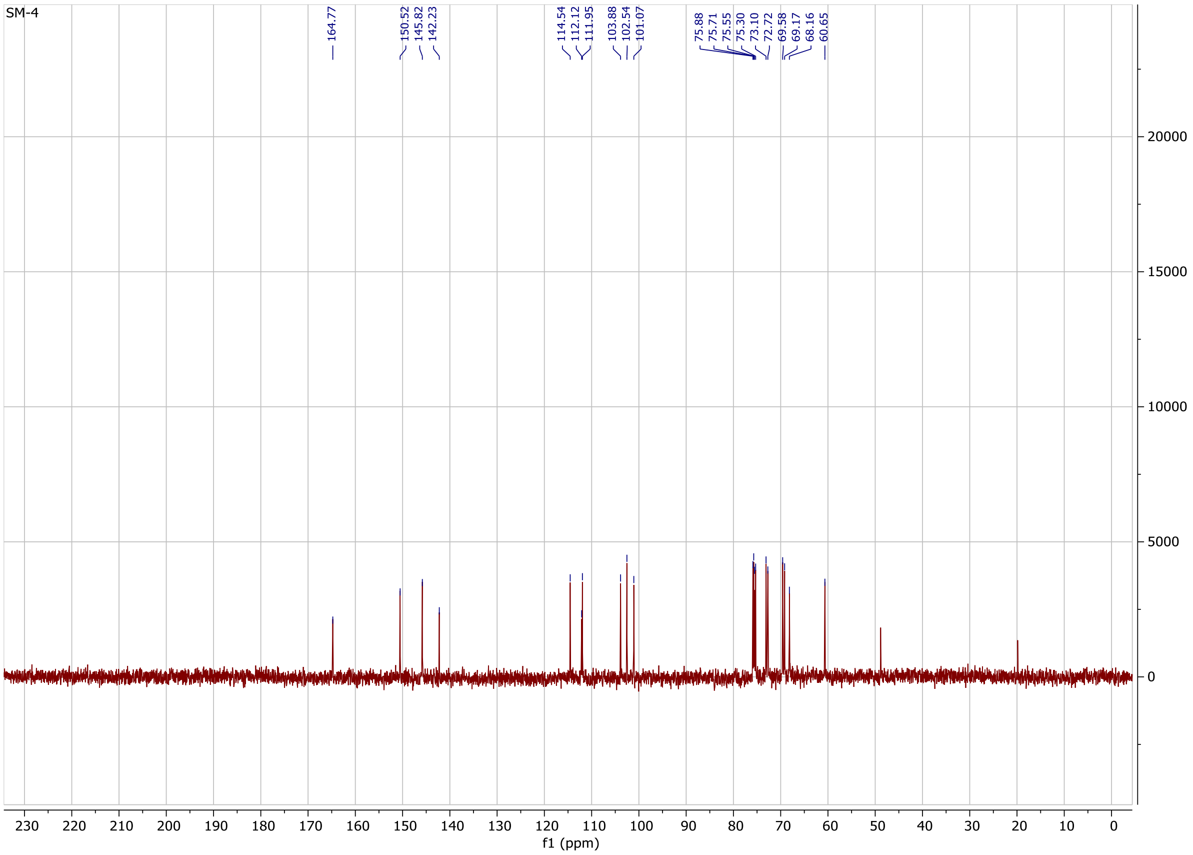

Figure S22. ^{13}C-NMR spectrum of compound **16**.

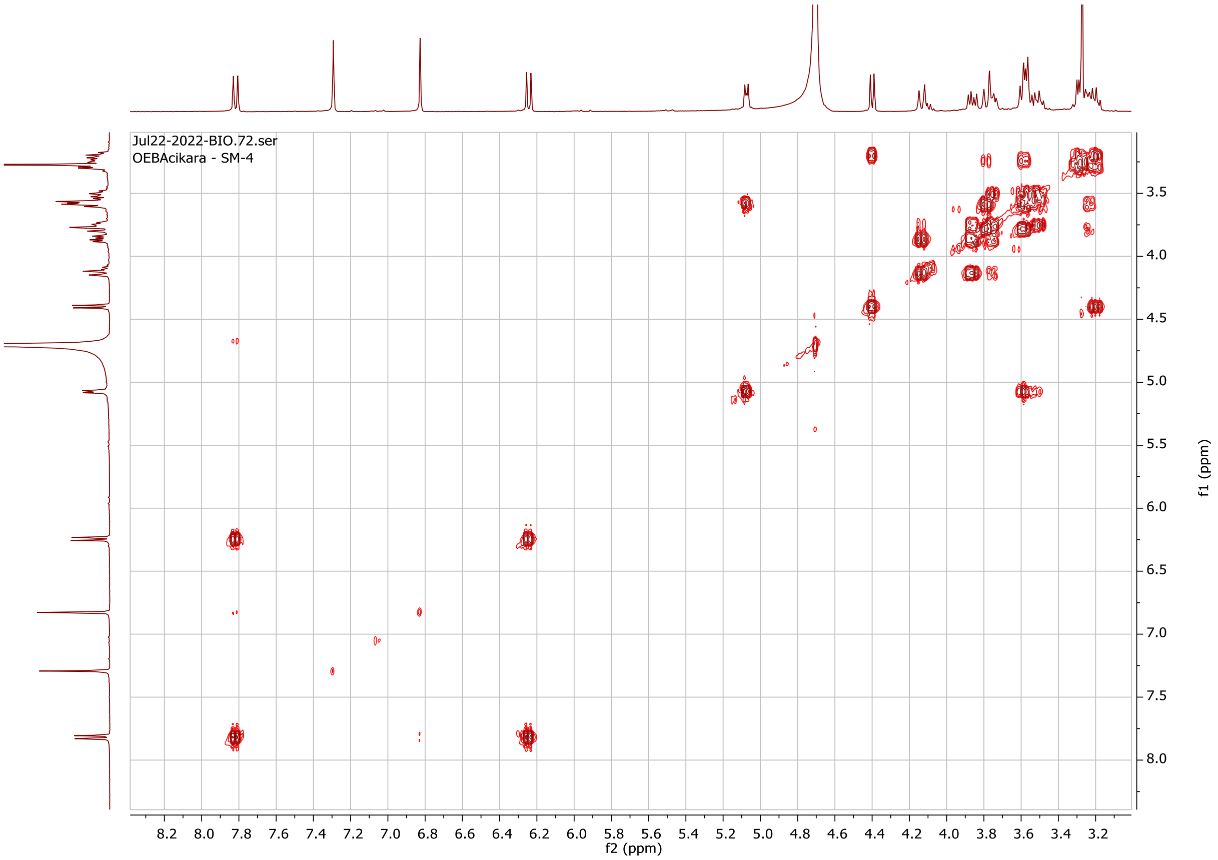

Figure S23. COSY-NMR spectrum of compound **16**.

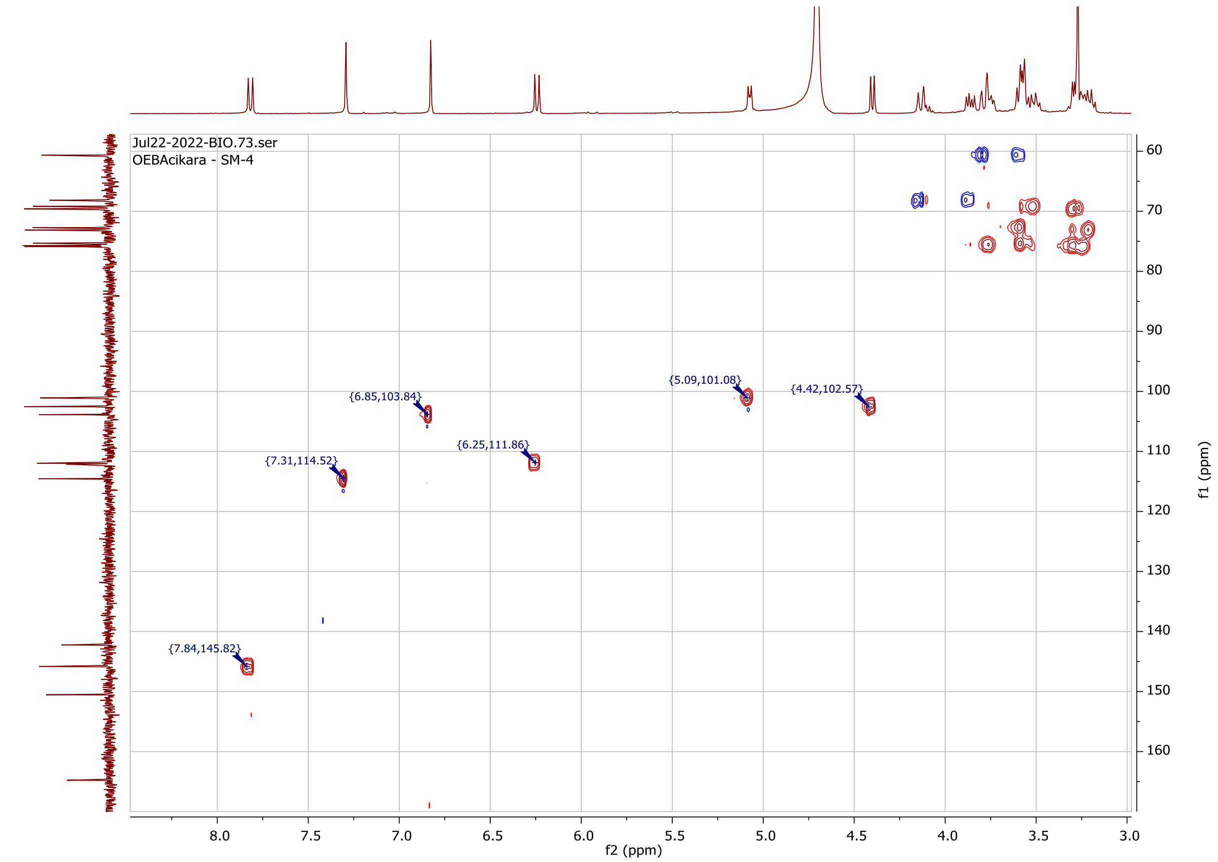

Figure S24. HSQC-NMR spectrum of compound **16** sugar part.

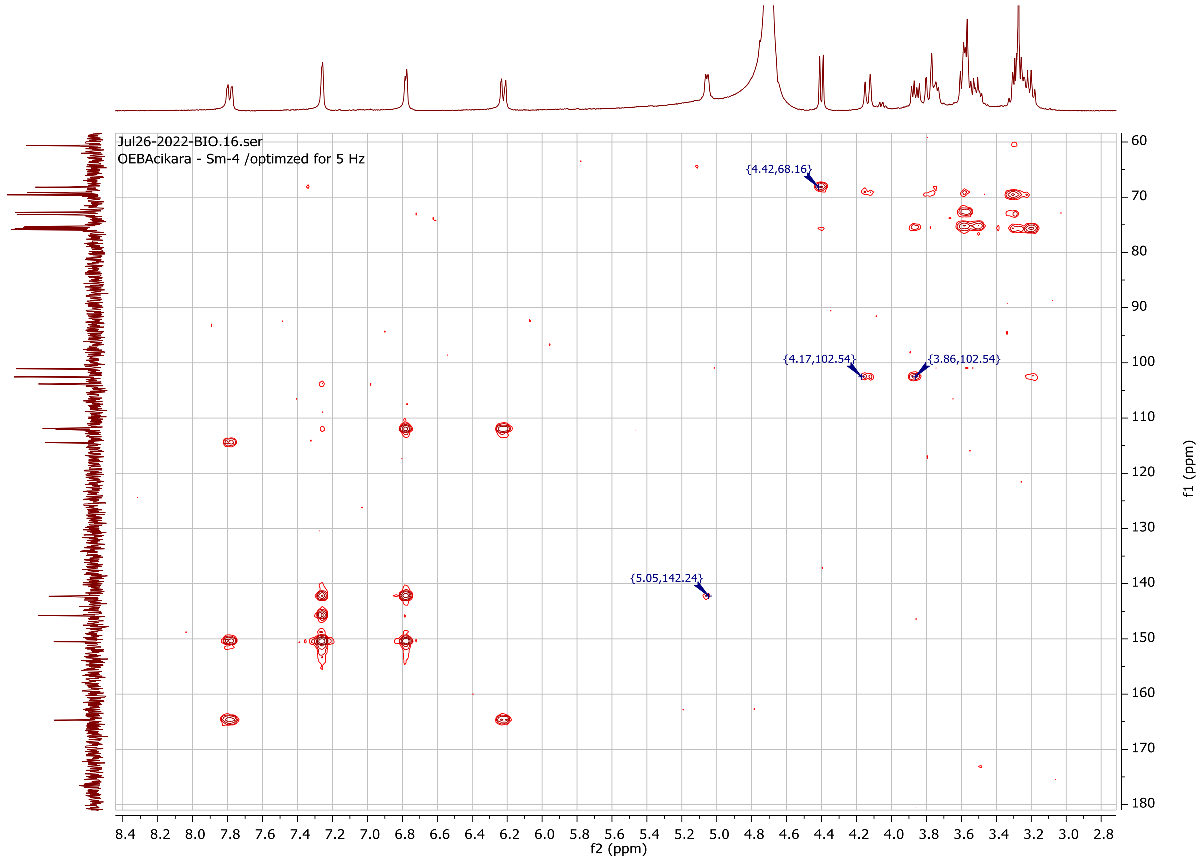

Figure S25. HMBC spectrum of compound **16** (sugar part HMBC correlations were assigned).

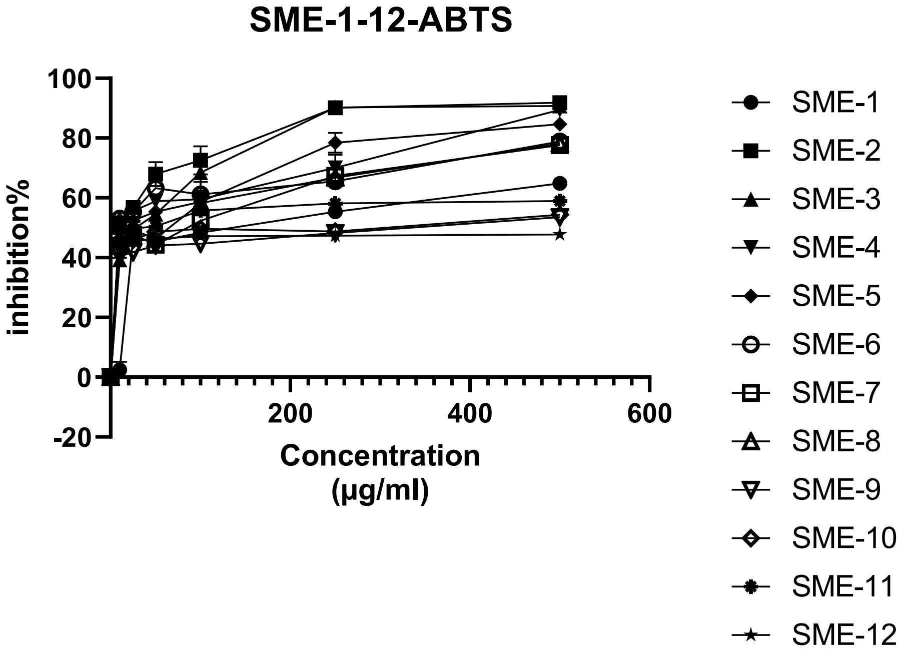

Figure 26. Calibration curve (ABTS) of *P. szowitsii* ethylacetate phase fractions.

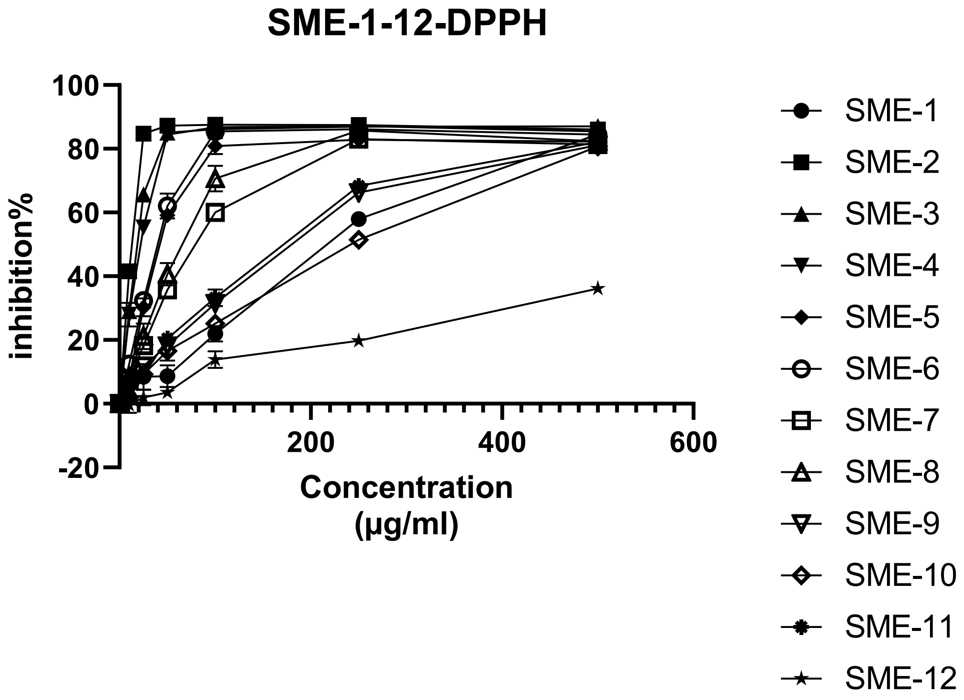

Figure 27. Calibration curve (DPPH) of *P. szowitsii* ethylacetate phase fractions.

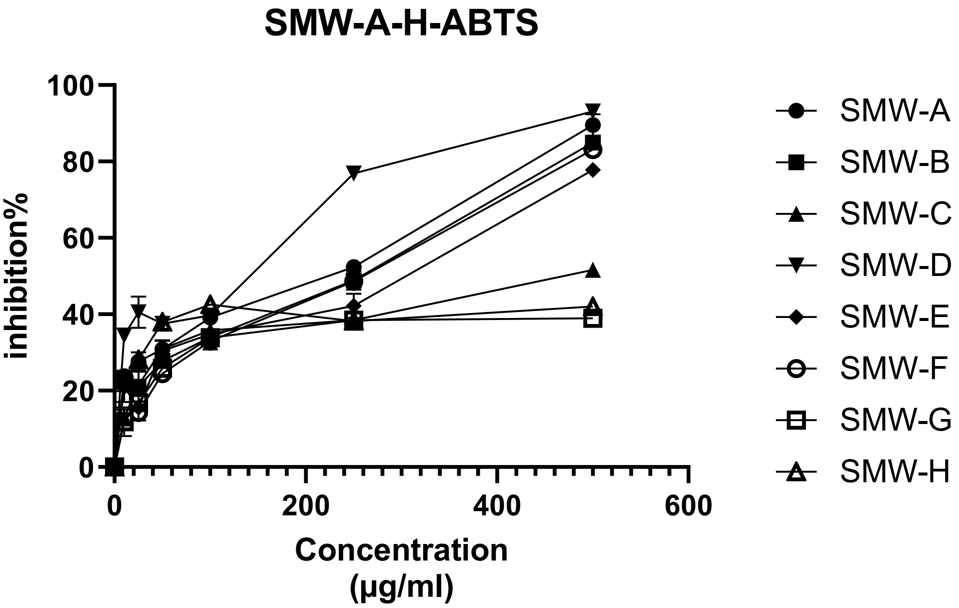

Figure 28. Calibration curve (ABTS) of *P. szowitsii* water phase fractions.

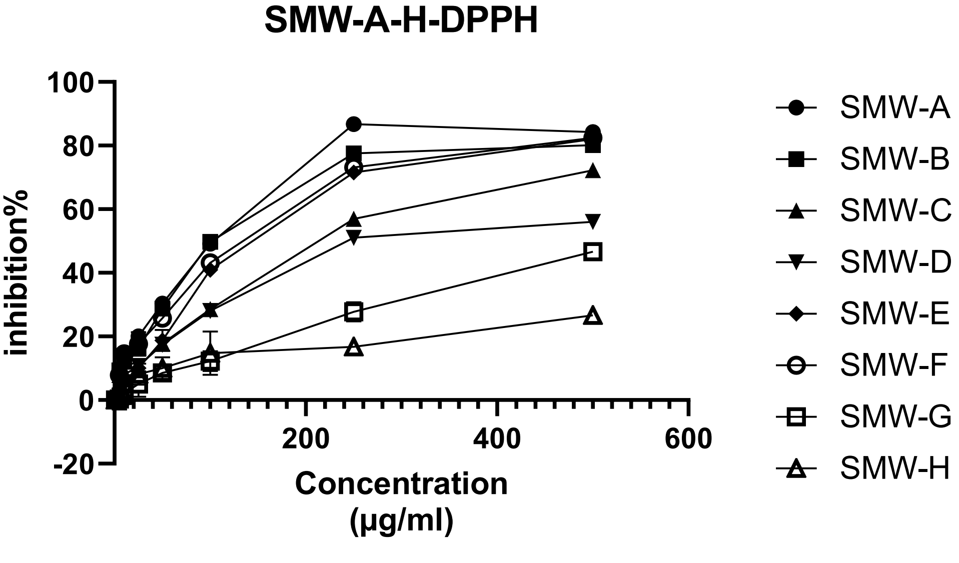

Figure 29. Calibration curve (DPPH) of *P. szowitsii* water phase fractions.

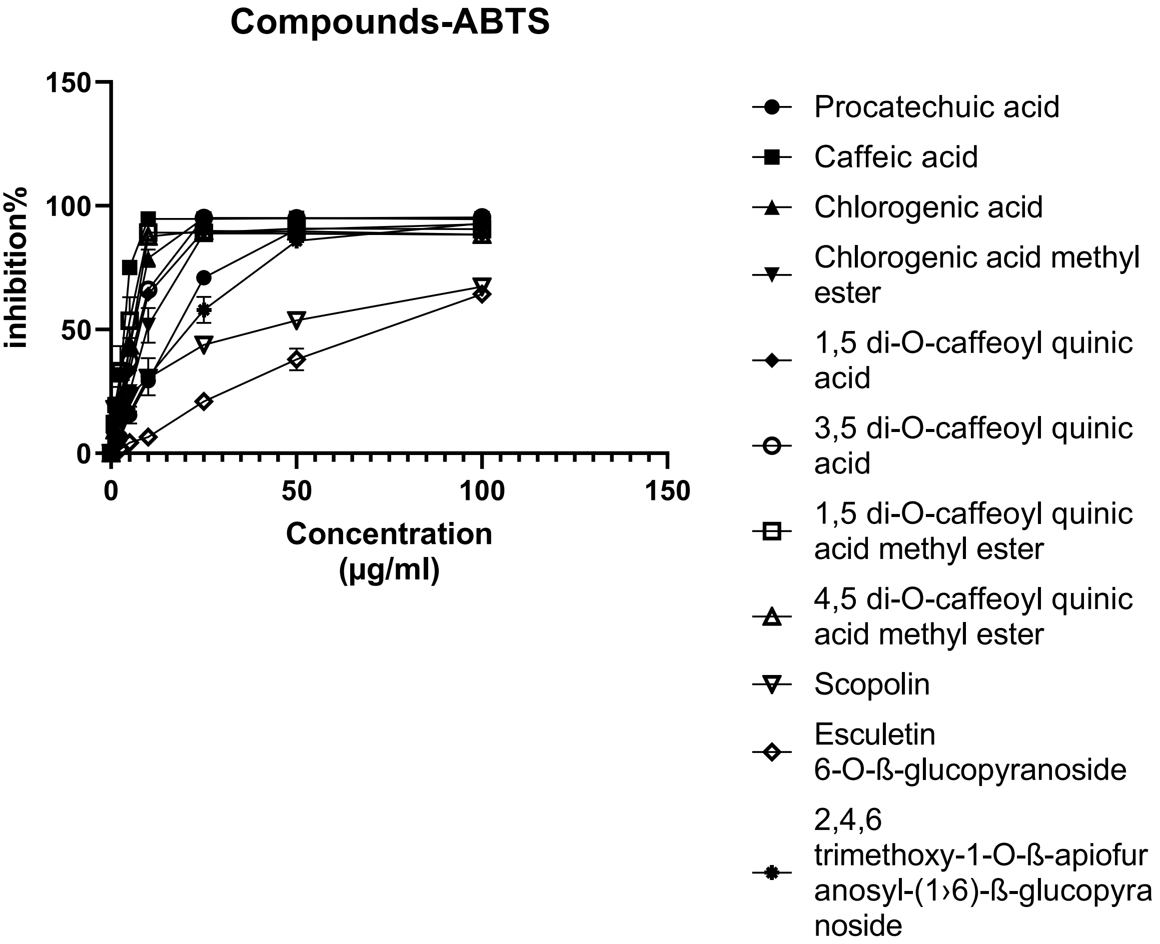

Figure 30. Calibration curve (ABTS) of compounds obtained from *P. szowitsii*.

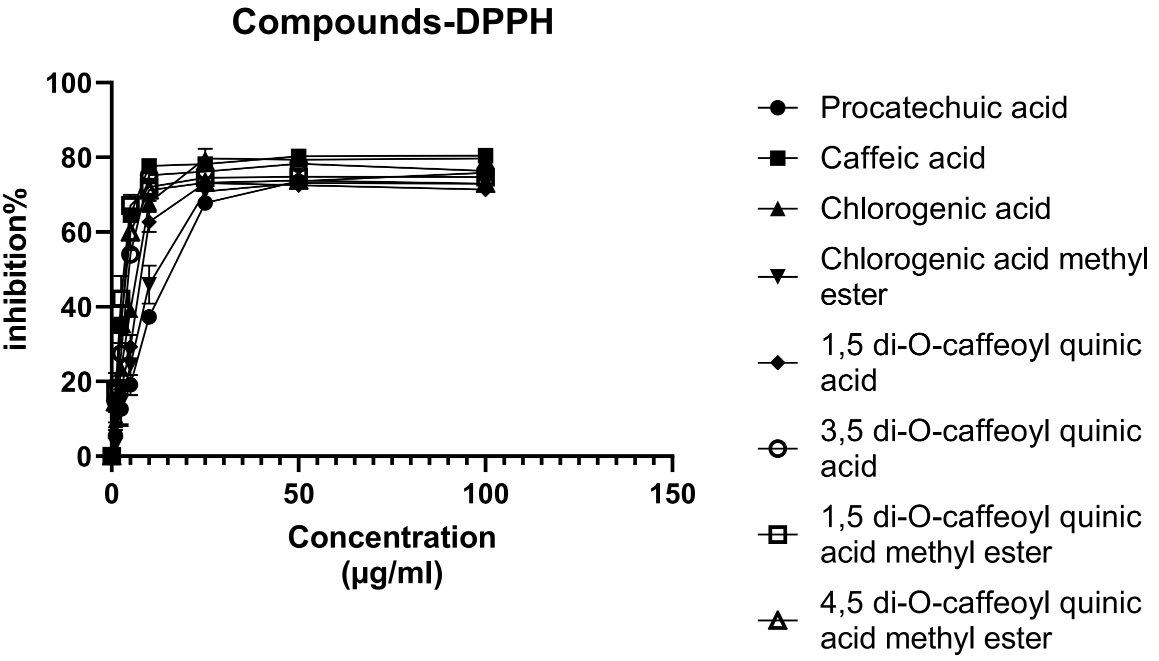

Figure 31. Calibration curve (DPPH) of compounds obtained from *P. szowitsii*.
